# Electronic Interaction at Cu–O–Ni Heterointerface Promotes Electrocatalytic Nitrate Reduction to Ammonia and Zinc‐Nitrate Battery

**DOI:** 10.1002/advs.202521252

**Published:** 2026-01-04

**Authors:** Taozhi Lv, Lekuan Yang, Can Hong, Yihua Zhu, Jianhua Shen, Chunzhong Li

**Affiliations:** ^1^ Shanghai Engineering Research Center of Hierarchical Nanomaterials Frontiers Science Center For Materiobiology and Dynamic Chemistry School of Materials Science and Engineering Key Laboratory for Ultrafine Materials of Ministry of Education East China University of Science and Technology Shanghai China

**Keywords:** ammonia synthesis, electrochemical nitrate reduction, heterostructure, interfacial synergy, Zinc‐nitrate battery

## Abstract

Electrochemical nitrate‐to‐ammonia conversion powered by sustainable green electricity is a promising supplement to the traditional Haber–Bosch process, but it remains limited by low NH_3_ yield and Faradaic efficiency (FE). Herein, we report the synthesis and performance of a Cu_2_O/Cu(OH)_2_@Ni(OH)_2_ heterostructure catalyst. The interface exploits strong electronic interactions between Cu and Ni species, promoting efficient NO_3_^−^ adsorption and accelerating in situ water dissociation for hydrogenation steps. In an H‐cell, the catalyst achieved an FE of 99.6 % with an NH_3_ yield of 1.14 mmol h^−1^ mg cat^−1^, while a flow electrolyzer maintained 97.9 % efficiency and 17.13 mmol h^−1^ mg cat^−1^ at −600 mA cm^−2^. Via density functional theory (DFT) calculations and in situ characterization, the interface uses strong Cu–O–Ni electronic interactions to boost efficient NO_3_^−^ adsorption and accelerate in situ water dissociation for hydrogenation steps. When integrated as a cathode into a Zn‐NO_3_^−^ hybrid battery, the material served as a high‐performance cathode. The resulting battery's open‐circuit voltage reached 1.45 V, while its power density peaked at 6.47 mW cm^−2^. The integrated device continuously produced 2.78 mg h^−1^ cm^−2^ of NH_3_ with 93.6 % FE and exhibited robust stability (<2 % voltage decay over 24 h).

## Graphical abstract

The strong Cu–O–Ni electronic interaction in the heterostructured catalyst can enhance the effective adsorption of NO_3_^−^ and accelerate the in situ hydrolysis dissociation of the hydrogenation step. The Zn‐NO_3_ hybrid battery assembled with this catalyst achieves an open‐circuit voltage of 1.45 V, a peak power density of 6.47 mW cm^−2^, and can continuously produce NH_3_ at a rate of 2.78 mg h^−1^ cm^−2^ with FE of 93.6 %.

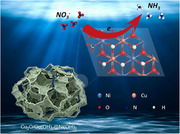

## 1 Introduction

Ammonia, the second top industrial chemical (mainly for fertilizers), is also a promising carbon‐free H_2_ energy carrier (17.6 wt. % H_2_) [1, 2, 3]. But its industrial production via fossil‐fuel‐based Haber–Bosch uses 2 % global energy and emits 1.5 % global CO_2_ [4, 5]. Electrochemical NH_3_ synthesis with clean energy is a better alternative (low fossil use, zero emissions). The electrochemical reduction of nitrate (NO_3_RR) is promising for this, but forms byproducts (NO_2_^−^, N_2_), lowering NH_3_ selectivity [6, 7]. Thus, NO_3_RR catalysts need to speed up NO_3_^−^ reduction and intermediate protonation for high NH_3_ selectivity.

Copper (Cu)‐based catalysts are extensively investigated due to their optimal d‐band center, which facilitates the adsorption and activation of nitrate (NO_3_) to nitrite (NO_2_^−^) [8, 9]. However, they often suffer from a kinetic mismatch: the subsequent reduction of NO_2_^−^ to NH_3_ is sluggish compared to the rapid initial step, leading to the accumulation of nitrite intermediates and limited ammonia selectivity [10]. Furthermore, operando studies have revealed that Cu species are susceptible to structural reconstruction and phase transformation under cathodic potentials, compromising long‐term stability [11]. On the other hand, Nickel (Ni)‐based materials (e.g., nickel hydroxides), while exhibiting weaker nitrate affinity, excel in water dissociation to supply active hydrogen species (H^*^), which are essential for the hydrogenation steps [12, 13]. Therefore, coupling Cu with Ni to construct heterostructures offers a compelling strategy. Such a design can synergize the nitrate‐activation capability of Cu with the hydrogen‐supplying ability of Ni, effectively bridging the kinetic gap and enhancing structural durability [14]. Heterostructure engineering has proven to be a powerful strategy for enhancing catalytic performance by creating synergistic interfaces that optimize the electronic properties of active sites [15, 16, 17]. In the realm of copper‐based catalysts for NO_3_RR, this approach has become a cornerstone of rational design, as the interface can induce charge redistribution to create electron‐deficient centers that facilitate NO_3_^−^ adsorption and activation [18, 19, 20].

Beyond enhancing nitrate adsorption, providing a sufficient supply of active hydrogen (H) from water splitting is also critical for the successful progression of NO_3_RR, and heterostructures are adept at creating bifunctional sites to co‐facilitate these processes. Li et al. designed and prepared a Cu/a‐CeO_x_ heterostructure by introducing an amorphous CeO_x_ support. This support was capable of providing sufficient *H and synergistically catalyzing the NO_3_RR [21]. Liu et al. found that introducing an amorphous CoO support to build the a‐CoO/Cu_2_O tandem catalyst allowed it to supply sufficient active hydrogen and synergistically catalyze NO_3_RR. They attributed this success to the heterojunction interface's synergistic cascade effect [22]. A common thread in these advanced catalysts is that a well‐designed heterostructure can address the primary challenges of NO_3_RR. It achieves this by creating electronically optimized centers for NO_3_^−^ binding while also promoting water dissociation, thereby providing a spatially coupled supply of reactants and protons to drive the reaction efficiently [23, 24, 25].

Recent work has translated half‐cell nitrate‐to‐ammonia catalysis into zinc‐nitrate (Zn‐NO_3_^−^) hybrid batteries, which compellingly combine wastewater denitrification with energy output [26, 27, 28, 29, 30]. In these devices, the NO_3_RR at the cathode is coupled to Zn oxidation at the anode, delivering higher open‐circuit voltages than Zn‐air batteries while avoiding O_2_ crossover limitations. Encouraging progress has been made on the cathode side, with advanced catalysts demonstrating high performance. For example, Yao et al. reported that optimizing the d‐band center of the Ru nodes boosted the power density of their Ru‐doped MOFs to 4.99 mW cm^−2^ [31]. Similarly, a high‐entropy hydroxide nanocatalyst delivered 3.62 mW cm^−2^ with nearly quantitative Faradaic efficiency (FE) [32]. Despite this immense potential, the practical realization of high‐performance, long‐lasting Zn‐NO_3_^−^ batteries is severely impeded by a confluence of deep‐seated electrochemical challenges that plague both the nitrate‐reducing cathode and the metallic zinc anode [33, 34, 35]. These issues are not isolated but are intricately interconnected, creating a complex web of degradation pathways that must be comprehensively understood and addressed.

Addressing this complex web of interconnected failure modes requires a multi‐pronged strategy, yet it is clear that improving the performance of the nitrate‐reducing cathode is the foundational first step. A highly efficient and selective cathode that can operate at a lower overpotential would not only boost the battery's power output but would also alleviate the electrochemical stress on the anode, thereby mitigating a primary driver of its degradation. With this pivotal goal in mind, herein, we report the successful fabrication of a Cu_2_O/Cu(OH)_2_@Ni(OH)_2_ heterostructure catalyst. The catalyst achieved a FE of 99.6 % and an NH_3_ yield of 1.14 mmol h^−1^ mg cat^−1^ in an H‐cell, while a flow electrolyzer maintained 97.9 % efficiency and 17.13 mmol h^−1^ mg cat^−1^ at −600 mA cm^−2^. Through density functional theory (DFT) theoretical calculations and in situ characterization, the interface leverages strong electronic interactions between Cu and Ni species, promoting efficient NO_3_^−^ adsorption and accelerating in situ water dissociation for the hydrogenation steps. Coupling the cathode with a 0.3 mm Zn anode yielded a Zn‐NO_3_^−^ battery that delivered 6.47 mW cm^−2^ peak power density while preserving 93.6 % efficiency and 2.78 mg h^−1^ cm^−2^ NH_3_ production. These findings establish that designing synergistic heterostructures is a robust strategy for high‐rate nitrate removal, green ammonia synthesis and simultaneous energy harvesting.

## 2 Results and Discussion

**FIGURE 1:**
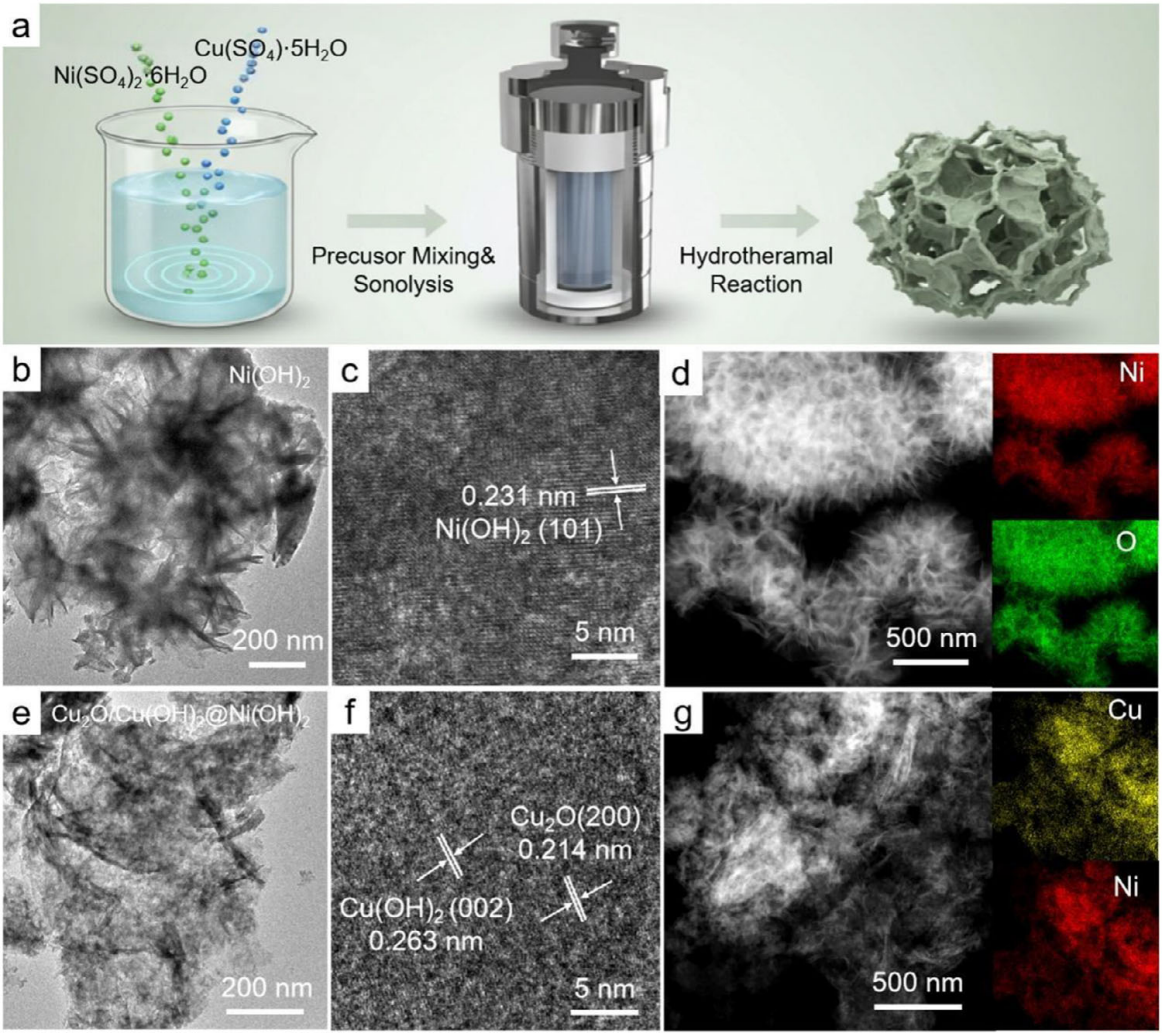
(a) Preparing the Cu_2_O/Cu(OH)_2_@Ni(OH)_2_ heterostructure via a one‐step hydrothermal method. TEM images of Ni(OH)_2_ at 200 nm (b), 5 nm (c) scales, and corresponding EDX element mappings (d). TEM images of Cu_2_O/Cu(OH)_2_@Ni(OH)_2_ at 200 nm (e), 5 nm (c) scales, and corresponding EDX element mappings (g).

The synthesis process of the Cu_2_O/Cu(OH)_2_@Ni(OH)_2_ heterostructure is schematically illustrated in Figure 1a. The catalyst was prepared via a one‐step hydrothermal method, and its initial characterization involved using transmission electron microscopy (TEM) to probe the morphology and microstructure. For comparison, the pristine Ni(OH)_2_ was characterized. As shown in Figure 1b, it exhibits a distinct needle‐like morphology, which is significantly different from the nanosheet structure of the heterostructure. A clear lattice fringe is visible in the HRTEM image in Figure 1c, exhibiting a d‐spacing of 0.231 nm, a value consistent with the (101) plane of Ni(OH)_2_. The energy dispersive X‐ray (EDX) mapping in Figure 1d confirms the uniform distribution of Ni and O throughout the needle‐like structure.

The overall morphology of the Cu_2_O/Cu(OH)_2_@Ni(OH)_2_ heterostructure is revealed in the low‐magnification TEM image Figure 1e, which shows a composition of stacked and wrinkled nanosheets. A detailed analysis of the HRTEM image in Figure 1f confirms the material's crystalline nature. Specifically, two distinct lattice spacings were measured at 0.263 and 0.214 nm, which can be attributed to the (002) plane of Cu(OH)_2_ and the (200) plane of Cu_2_O, respectively [36, 37]. This confirms the coexistence of both copper species within the heterostructure. The elemental distribution was then mapped using EDX in Figure 1g. The maps for Cu, Ni, and O show a uniform distribution of all three elements across the nanosheet structure, suggesting a homogeneous integration at the nanoscale without obvious phase separation. We further examined the morphology of the Cu_2_O/Cu(OH)_2_@Ni(OH)_2_ catalyst via scanning electron microscopy (SEM), and representative images are presented in Figure S1. A stark contrast was observed in the morphology and crystal structure when comparing the Cu_2_O/Cu(OH)_2_@Ni(OH)_2_ heterostructure to the pristine Ni(OH)_2_. This difference strongly suggests that the introduction of copper during the hydrothermal synthesis fundamentally alters the material's growth and formation process.

**FIGURE 2:**
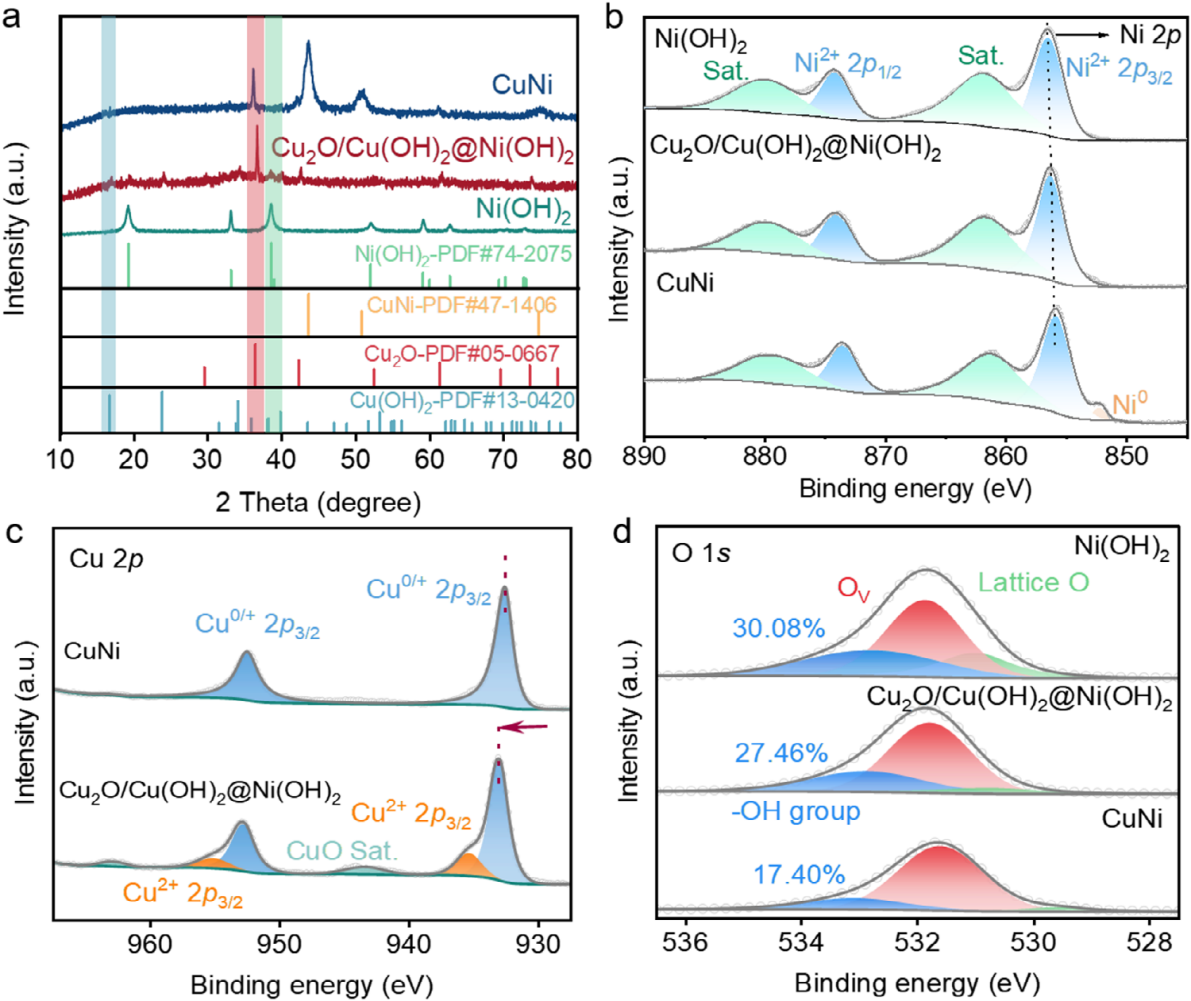
Structural and chemical state characterization of the catalysts. (a) XRD patterns of the as‐prepared materials. High‐resolution XPS spectra for the Ni 2*p* (b), Cu 2*p* (c) and O 1*s* (d) regions of Cu_2_O/Cu(OH)_2_@Ni(OH)_2_, Ni(OH)_2_ and CuNi.

This structural transformation was further corroborated by X‐ray diffraction (XRD) analysis. As shown in Figure 2a, compared to the synthesized Ni(OH)_2_, the XRD pattern of the heterostructure clearly exhibits the characteristic diffraction peaks corresponding to Cu(OH)_2_ (JCPDS: 13–0420) and Ni(OH)_2_ (JCPDS: 74–2075), in addition to the Cu_2_O phase. We note that the diffraction intensities of the hydroxide phases are relatively weak compared to the oxide phase. This is attributed to the low crystallinity of the hydroxides formed during the hydrothermal reaction and the lattice distortion induced by the strong interfacial interactions between the Cu and Ni species. This result confirms the successful coexistence of Cu_2_O, Cu(OH)_2_, and Ni(OH)_2_ in the heterostructure [38]. Furthermore, subsequent calcination of the heterostructure under a 5 % H_2_/Ar atmosphere yielded a structure similar to CuNi alloys (JCPDS: 47–1406) [39]. This result is also consistent with the TEM results mentioned above.

X‐ray photoelectron spectroscopy (XPS) provided further insights into the chemical states of the elements within the Cu_2_O/Cu(OH)_2_@Ni(OH)_2_ catalyst. As expected, the survey spectrum revealed the presence of Cu, Ni, and O in Figure S2. The high‐resolution spectrum for the Ni 2*p* region is subsequently detailed in Figure 2b. The deconvoluted spectra clearly display the characteristic spin‐orbit doublets of Ni^2+^ (2*p*_3/2_ and 2*p*_1/2_) and their corresponding satellite peaks. Compared to pristine Ni(OH)_2_, the Ni 2*p* binding energies in the heterostructure exhibit a negative shift, suggesting an increase in electron density around the Ni centers. This indicates an electron transfer from Cu to Ni species at the interface. After calcination in a H_2_/Ar atmosphere to form the CuNi alloy, the binding energy shifted even further, and the Ni° content increased, confirming enhanced reduction.

Conversely, the electron‐deficient nature of Cu in the heterostructure was verified by its 2*p* spectrum (Figure 2c). Deconvolution of this spectrum revealed two primary components: peaks centered at 933.1 and 935.5 eV, which are attributed to the presence of Cu^2+^ and Cu^+^ species, respectively [40]. This positive shift in binding energy compared to the CuNi alloy (where Cu^+^ is at 932.7 eV) confirms that Cu species in the heterostructure are electron‐deficient. This bidirectional electron transfer between Cu and Ni, creating electron‐deficient Cu sites and electron‐rich Ni sites, is a hallmark of the strong electronic interactions at the heterostructure interface. The O 1*s* spectrum in Figure 2d was deconvoluted to better understand the surface chemistry. This analysis resolved three primary components with peaks centered at 529.5, 531.8, and 533.1 eV, which were assigned to lattice oxygen (M─O), oxygen vacancies (O_v_), and hydroxyl groups (M─OH), respectively. A notable trend was observed in the relative content of surface hydroxyl groups. The pristine Ni(OH)_2_ exhibited the highest concentration at 30.08 %, which then reduced to 27.46 % for the Cu_2_O/Cu(OH)_2_@Ni(OH)_2_ heterostructure and further diminished to 17.4 % for the CuNi alloy. The abundance of hydroxyl groups at the interface, in synergy with the electronically modulated metal sites (electron‐deficient Cu and electron‐rich Ni), is expected to play a crucial role in the electrocatalytic process. Specifically, the electron‐deficient Cu sites can facilitate NO_3_^−^ adsorption, while the adjacent hydroxyl groups can promote water dissociation to provide protons for the hydrogenation steps.

**FIGURE 3:**
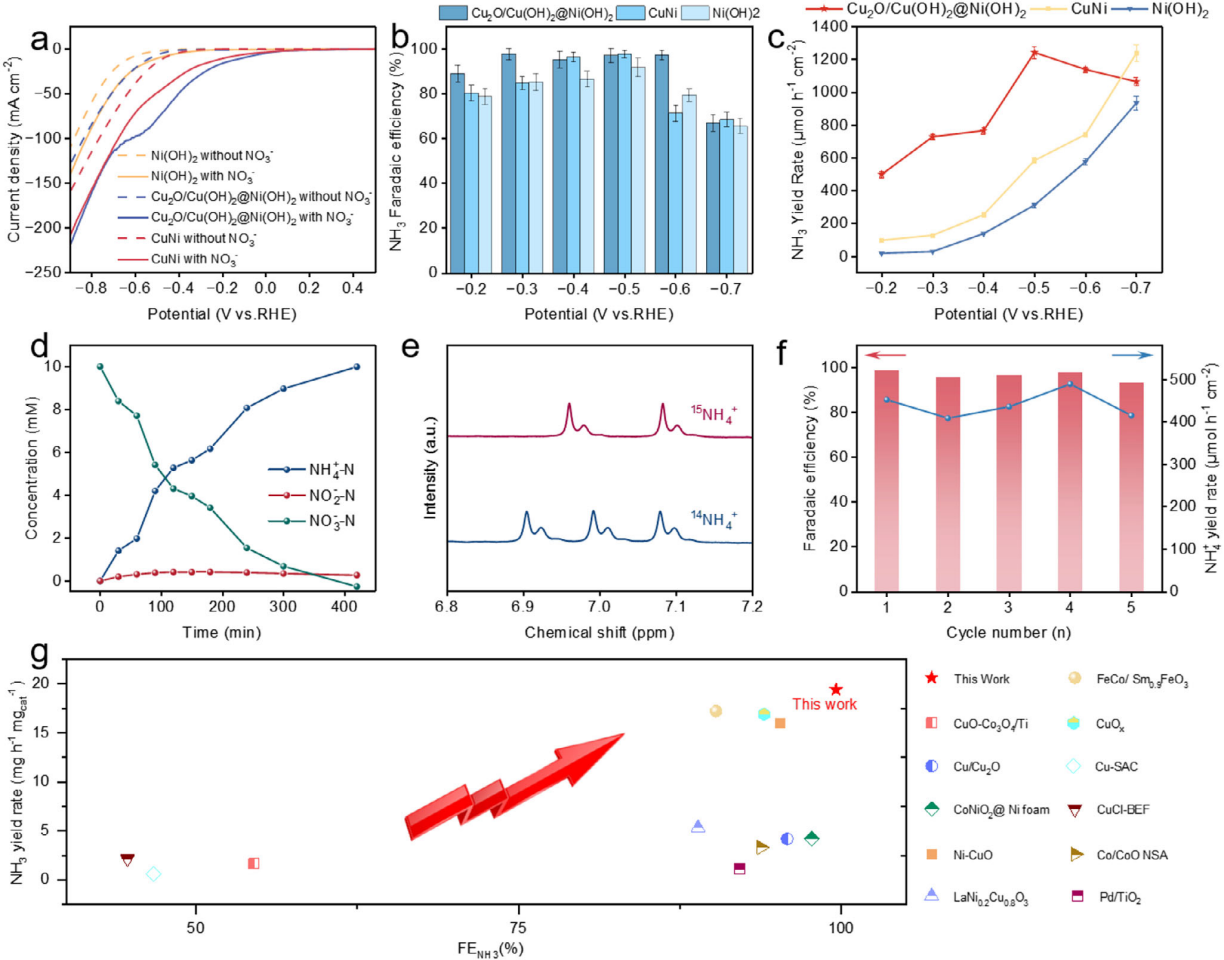
Electrochemical NO_3_RR performance. (a) LSV curves of Cu_2_O/Cu(OH)_2_@Ni(OH)_2_, CuNi, and Ni(OH)_2_ recorded in 1 m KOH and 0.1 m KNO_3_ electrolyte (without iR correction). Comparison of the potential‐dependent FEs (b) and NH_3_ yield rates (c) for the three catalysts. (d) Concentration profiles of NO_3_^−^‐N, NO_2_^−^‐N, and NH_3_^−^N over time during electrolysis with the Cu_2_O/Cu(OH)_2_@Ni(OH)_2_ catalyst at −0.5 V vs. RHE. (e) ^1^H NMR analysis of the electrolyte after electrolysis using ^15^NO_3_^−^ and ^14^NO_3_^−^ confirming NH_3_ production. (f) Cycling stability test of the Cu_2_O/Cu(OH)_2_@Ni(OH)_2_ catalyst, showing performance over five 2 h cycles at −0.5 V vs. RHE. (g) Benchmarking of the Cu_2_O/Cu(OH)_2_@Ni(OH)_2_ catalyst's FE and NH_3_ yield against other recently reported electrocatalysts.

To evaluate their NO_3_RR activities, we subsequently tested the Cu_2_O/Cu(OH)_2_@Ni(OH)_2_, CuNi, and Ni(OH)_2_ catalysts. All electrochemical measurements were conducted in an H‐type electrolytic cell containing a 1 m KOH electrolyte. We first performed linear sweep voltammetry (LSV) measurements under Ar‐saturated conditions to probe the catalysts' electrochemical behavior in Figure 3a. The tests were conducted both in the absence and presence of 0.5 m NO_3_^−^. The conductivity of the catalysts was found to be somewhat enhanced, and then the formation of CuNi alloy after calcination and reduction of Cu_2_O/Cu(OH)_2_@Ni(OH)_2_ further enhanced the electrical conductivity of the catalyst. Notably, a prominent reduction wave appears in the range of −0.4 to −0.6 V vs. RHE for the Cu_2_O/Cu(OH)_2_@Ni(OH)_2_ catalyst, which is attributed to the diffusion‐limited reduction of accumulated nitrite intermediates (NO_2_^−^) stemming from the kinetic mismatch in the cascade reaction steps [41]. The current densities of the catalysts all increased, and the current densities of Cu_2_O/Cu(OH)_2_@Ni(OH)_2_ and CuNi increased more under the same potential, indicating that the presence of Cu is an important factor in the enhancement of the performance of the NO_3_RR. However, compared to the CuNi catalyst, the Cu_2_O/Cu(OH)_2_@Ni(OH)_2_ catalyst exhibits a lower overpotential at the same current density, indicating superior NO_3_RR reactivity. This suggests that the electron‐deficient Cu sites, created by the heterostructure interface, enhance the catalytic performance.

The electrocatalytic NO_3_RR performance was assessed via controlled‐potential electrolysis for 1 h within a potential window of −0.2 to −0.7 V vs. RHE. The liquid products, particularly generated ammonia (NH_3_), were quantified using the indophenol blue colorimetric method via UV–vis spectrophotometry, with the corresponding results for all three catalysts compiled in Figure 3b. Crucially, the accuracy of the colorimetric quantification for NH_3_ was rigorously verified by quantitative ^1^H Nuclear Magnetic Resonance (NMR) spectroscopy (Figure S3). In addition, we have supplemented the Supporting Information with standard calibration curves for nitrite and ammonia (using both UV–vis and NMR methods), as shown in Figures S4–S6. All curves exhibit excellent linearity (R^2^ >0.999), ensuring reliable quantification. Regarding nitrate conversion and selectivity, we strictly followed the standard dual‐wavelength method (220 and 275 nm) and Equations S1–S5 described in the Experimental Section to eliminate organic interference. The performance of the three catalysts prepared in the selected potential intervals exhibited a typical volcano‐like trend, while Cu_2_O/Cu(OH)_2_@Ni(OH)_2_ exhibited the highest FE and yield, reaching up to 99.6 % and 1.14 mmol h^−1^ mg_cat_^−1^ at −0.6 V vs. RHE (Figure 3c), and up to 1.25 mmol h^−1^ mg_cat_^−1^ at −0.5 V vs. RHE, which has surpassed that of most catalysts that have been reported. Further confirmation of the reaction progression was obtained from the time‐dependent concentration profiles at −0.5 V vs. RHE (Figure 3d), which reveal a continuous conversion of nitrate to ammonia with minimal nitrite accumulation. Additionally, isotope labeling experiments using ^15^NO_3_^−^ (Figure 3e) displayed a characteristic doublet for ^15^NH_4_^+^, rigorously verifying that the detected ammonia originates from the nitrate reduction. The FE of NO_3_RR decreased with the increasing current density; this decline is primarily attributed to the competing hydrogen evolution reaction (HER), which becomes more pronounced as the potential shifts. To provide a comprehensive view of the product selectivity, the FE distribution of all detected products (NH_3_, NO_2_^−^, and H_2_) is presented in Figure S7. The quantitative analysis reveals that nitrite formation is effectively suppressed across the potential range. While hydrogen evolution is negligible at lower overpotentials, its contribution increases slightly at −0.7 V vs. RHE, which aligns with the observed decrease in ammonia efficiency. Although the NO_3_RR activity of Cu_2_O/Cu(OH)_2_@Ni(OH)_2_ decreased relatively weakly compared with the significant decrease of CuNi and Ni(OH)_2_ reactivities, and had more than 90 % of FE over a wide range of potentials, which confirms that this catalyst had a stronger inhibitory effect on HER.

The enhanced NO_3_RR activity of the Cu_2_O/Cu(OH)_2_@Ni(OH)_2_ catalyst prompted an investigation into its electrochemically active surface area (ECSA). We estimated the ECSA by calculating the double‐layer capacitance (C_dl_), a value directly proportional to the active surface area. To determine the C_dl_, we performed cyclic voltammetry (CV) at various sweep speeds within the non‐Faradaic potential region (Figure S8). Based on these measurements, the calculated C_dl_ values were 0.23, 0.12, and 0.018 mF cm^−2^ for the Cu_2_O/Cu(OH)_2_@Ni(OH)_2_, CuNi, and Ni(OH)_2_ catalysts, respectively. The significantly larger C_dl_ value of Cu_2_O/Cu(OH)_2_@Ni(OH)_2_ indicates that it possesses the largest ECSA of the three catalysts. This finding suggests that the introduction of Cu into the Ni(OH)_2_ structure is a crucial factor in exposing more active sites. This increased surface area, in synergy with the appropriate hydroxyl group coverage and the unique electronic properties of the heterostructure, is highly favorable for NO_3_^−^ adsorption and subsequent reduction. We then employed electrochemical impedance spectroscopy (EIS) to probe the charge transfer kinetics of the catalysts. The resulting Nyquist plots (Figure S9) revealed that both the Cu_2_O/Cu(OH)_2_@Ni(OH)_2_ heterostructure and the CuNi alloy possess a significantly smaller charge transfer resistance (R_ct_) than pristine Ni(OH)_2_. This finding suggests that the incorporation of copper enhances the material's conductivity, creating an efficient pathway for electron transport. Ultimately, this accelerated charge transfer kinetics is a key factor in boosting the overall Ni(OH)_2_ activity.

In addition to excellent reactivity, the stability of a good catalyst is the basis for long‐term operation. The stability of Cu_2_O/Cu(OH)_2_@Ni(OH)_2_ in 0.1 m KNO_3_ reactant was tested for five consecutive cycles, and the potential with the highest yield −0.5 V vs. RHE was chosen as the reaction potential, and the yield and FE are shown in Figure 3f, from which it can be seen that the catalyst's FE and yield fluctuated within a small range over the course of five cycles, and the FE was always above 93 %. The structure of the catalyst after the cyclic stability test was determined by XRD and XPS analysis. The post‐reaction XRD pattern (Figure S10) exhibits diffraction peaks consistent with the fresh catalyst, confirming that the crystallographic structure remains intact without phase transformation into metallic Cu. Additionally, the valence states of Cu and Ni (Figure S11) in the XPS pattern remained basically unchanged compared to those before the reaction, which implies that the catalyst can maintain good structural stability during the long‐time electroreduction process. This indicates that this catalyst has good cycling stability, and the long‐term operation of the catalyst is feasible.

Relative to control samples and other recently reported NO_3_RR catalysts, the Cu_2_O/Cu(OH)_2_@Ni(OH)_2_ composite demonstrated superior NH_3_ FE and production yield (Figure 3g; Table S1). Several synergistic effects arising from the unique heterostructure of the Cu_2_O/Cu(OH)_2_@Ni(OH)_2_ catalyst are responsible for its superior NO_3_RR performance. First, the introduction of Cu enhances the electrical conductivity and increases the electrochemically active surface area, exposing more active sites. Second, as revealed by XPS, strong electronic interactions at the interface create electron‐deficient Cu sites and an abundance of hydroxyl groups. These two components work in synergy: the electron‐deficient Cu sites facilitate NO_3_^−^ adsorption, while the adjacent ─OH groups promote water dissociation. This synergistic action accelerates the formation of key hydrogenation intermediates and thereby improves the selectivity to NH_3_.

**FIGURE 4:**
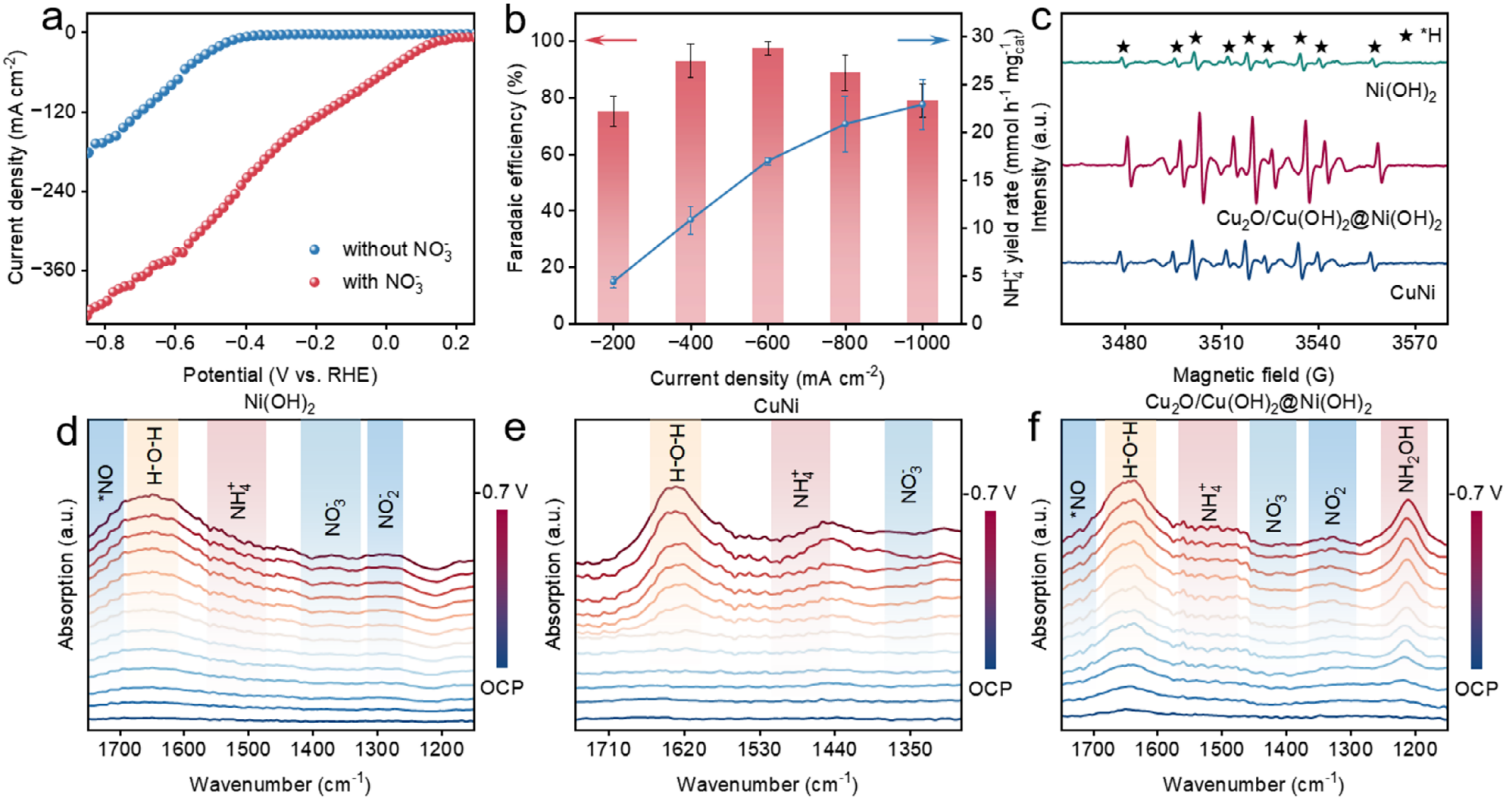
(a) LSV polarization curve for the Cu_2_O/Cu(OH)_2_@Ni(OH)_2_ catalyst recorded in a flow cell, the electrolyte was a mixture of 1 m KOH and 0.5 m KNO_3_^−^ (without iR‐compensated). (b) FE and yield of Cu_2_O/Cu(OH)_2_@Ni(OH)_2_ catalyst after electrochemical reduction for 1 h at different current densities in a flow cell. (c) Quasi in situ EPR analysis to detect active hydrogen (^.^H) species. The spectra were collected for all three catalysts in the presence of DMPO as a spin‐trap agent, but in the absence of NO_3_^−^, after 10 min of electrolysis at −0.5 V vs. RHE. Ni(OH)_2_ (d), CuNi (e), and Cu_2_O/Cu(OH)_2_@Ni(OH)_2_ (f) during the NO_3_RR electrolysis.

In order to further highlight its potential for industrial production, the electrochemical ammonia synthesis performance of NO_3_RR was tested at high current densities, using the improved mass transfer process of a flow cell reactor, so that the NH_3_ yield could be increased at high and stable current densities. To assess the catalyst's potential for industrial applications, we evaluated its NO_3_RR performance at high current densities using a flow cell with an electrolyte flow rate of 20 mL min^−1^ [42, 43]. This high flow rate serves to effectively dissipate the Joule heat generated during electrolysis, ensuring a stable reaction temperature even at high current densities. The detailed configuration is presented in Figure S12, which comprises cathode and anode chambers separated by a Nafion 117 membrane, with a working electrode geometric area of 1 cm^2^. As shown in the LSV polarization curves (Figure 4a), the flow cell configuration led to a significant advancement in the onset potential compared to the H‐type cell. Moreover, it enabled a much steeper increase in current density at equivalent potentials. We subsequently conducted further analyses across a wide current density range of −200 to −1000 mA cm^−2^. The corresponding potentials required to achieve these densities are detailed in Figure S13. These tests revealed that the maximum FE was attained at higher current densities under these conditions. Specific results are presented in Figure 4b, where the Cu_2_O/Cu(OH)_2_@Ni(OH)_2_ catalyst showed a maximum FE of 97.9 % for NH_3_ at −600 mA cm^−2^ with an ammonia yield of 17.13 mmol h^−1^ mg_cat_^−1^.

Due to the presence of an ongoing hydrogenation step in the NO_3_RR process, the proton H (*H) content directly affects the kinetics of the hydrogenation reaction of NO_3_RR. To probe the ability of the three catalysts to cleave H_2_O and generate active hydrogen (^.^H), we performed electron paramagnetic resonance (EPR) spectroscopy. In these tests, 5,5‐dimethyl‐1‐pyrroline n‐oxide (DMPO) was employed as a spin‐trapping agent for the H radical [44, 45]. The results are shown in Figure 4c, in which Cu_2_O/Cu(OH)_2_@Ni(OH)_2_ exhibits the strongest DMPO‐H signal. This reveals that the synergistic interface of the heterostructure, likely involving both the electron‐rich Ni sites and adjacent hydroxyl groups, significantly promotes H_2_O dissociation to generate active hydrogen. The DMPO‐H signal for the CuNi catalyst, however, was weaker. We attribute this decrease in signal intensity to the removal of surface hydroxyl groups, a consequence of the high‐temperature calcination process. The loss of these hydroxyl groups disrupts the crucial synergy between the metal sites and adjacent ─OH moieties. This synergy is essential for promoting efficient water splitting.

We employed in situ attenuated total reflection surface‐enhanced infrared absorption spectroscopy (ATR‐SEIRAS) to probe the reaction pathways and identify key intermediates formed on the catalyst surfaces during the NO_3_RR process. The resulting potential‐dependent spectra for the pristine Ni(OH)_2_ catalyst are presented in Figure 4d. As the potential becomes more negative, weak bands corresponding to adsorbed NO_3_^−^ and NO_2_^−^ intermediates can be observed, along with a minor peak for the final product, NH_3_. This indicates that Ni(OH)_2_ possesses some activity for nitrate reduction, though the accumulation of intermediates is not significant. For the CuNi alloy, the spectra in Figure 4e show a more pronounced peak for adsorbed NH_3_, suggesting that the presence of copper significantly enhances the conversion to the final product. This is consistent with the hypothesis that Cu serves as the primary active center for the deep reduction of nitrate. However, the signals for other hydrogenated intermediates remain weak.

Most notably, the Cu_2_O/Cu(OH)_2_@Ni(OH)_2_ heterostructure exhibits a unique spectral evolution (Figure 4f); the positive adsorption can be attributed to deoxygenation intermediates (*NO, *NO_2_), hydrogenation intermediates (NH_2_OH), and generated NH_3_ [46, 47]. Similar to the CuNi alloy, it shows a strong adsorption peak for the product NH_3_. Crucially, a very distinct and intense peak corresponding to the NH_2_OH intermediate emerges and strengthens at negative potentials. The prominent appearance of this key hydrogenated intermediate is a clear indicator of an efficient hydrogenation pathway on the heterostructure's surface. This observation strongly suggests that the synergistic interface, comprising electron‐deficient Cu sites and abundant hydroxyl groups, not only facilitates NO_3_^−^ adsorption but also excels at dissociating water to provide a steady supply of active hydrogen (H) for the subsequent hydrogenation steps. This efficient generation and utilization of intermediates ultimately drives the high selectivity and activity for ammonia synthesis.

**FIGURE 5:**
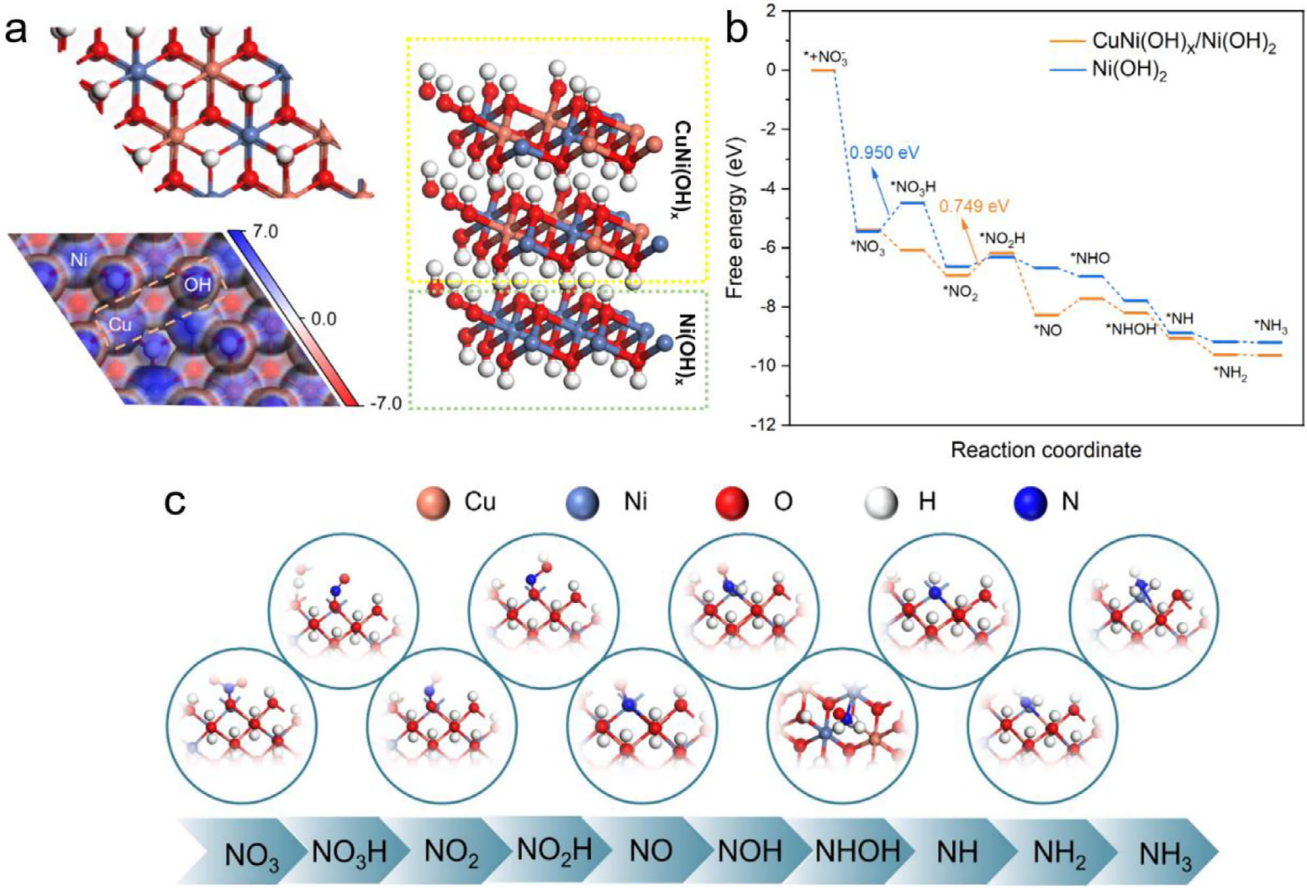
DFT calculations for the NO_3_RR on the Cu_2_O/Cu(OH)_2_@Ni(OH)_2_ surface. (a) The optimized geometric structure alongside its corresponding electrostatic potential surface. The electron density isosurface is set at 0.2 e bohr^−3^, with the electrostatic potential indicated by the color scale. (b) Calculated free energy diagram for the entire NO_3_RR pathway. (c) Adsorption configurations of key reaction intermediates.

A heterostructure model of the Cu_2_O/Cu(OH)_2_@Ni(OH)_2_ catalyst, depicted in Figure 5a, was constructed to gain deeper theoretical insights into the catalytic process of NO_3_RR. Based on this model, we plotted electron density isosurfaces. At the heterointerfaces, Cu and Ni sites exhibit positive electrostatic potentials, while adjacent ─OH groups, acting as Lewis sites, show significant negative electrostatic potentials. According to the color scale, Cu atoms connected to Ni display more positive, confirming the existence of charge transfer between Cu and Ni, which is also consistent with XPS analysis. Consequently, electron‐rich O atoms and electron‐deficient H atoms in H_2_O molecules tend to undergo targeted adsorption and dissociation into *H at Cu and ─OH sites. DFT calculations were performed on the reaction pathway of Cu_2_O/Cu(OH)_2_@Ni(OH)_2_ during NO_3_RR, as shown in Figure 5b. In situ ATR‐SEIRAS measurements identified the specific reaction pathway of NO_3_RR, with positive adsorption peaks observed for *NO_2_, *NO, and *NH_2_OH. Thus, the most probable reaction pathway is presented in Figure 5c. NO_3_^−^ is first adsorbed and released to form *NO_3_, which then undergoes protonation to generate *NO_3_H [48, 49]. Subsequent proton attack releases H_2_O, yielding *NO_2_. The sequence of hydrogenation/dehydration cycles for *NO_2_ reduction is: *NO_2_H → *NO → *NOH → *NHOH → *NH → *NH_2_ → *NH_3_. To elucidate the enhanced activity, the free energy profile of the pristine Ni(OH)_2_ catalyst was also calculated for comparison (blue line in Figure 5b). For Ni(OH)_2_, the potential‐determined step (PDS) is the protonation of NO_3_ to NO_3_H, with a high energy barrier of 0.950 eV. In contrast, for the heterostructure, the PDS shifts to the conversion of ^*^NO_2_ to ^*^NO_2_H, requiring a significantly reduced energy barrier of 0.749 eV. This substantial decrease in the energy barrier (0.201 eV) confirms that the Cu–O–Ni interface effectively facilitates the hydrogenation kinetics. Notably, this energy barrier is significantly lower than that of the competing HER, which stands at 0.847 eV (Figure S14). Therefore, upon application of electrical energy, the NO_3_RR process is favored.

**FIGURE 6:**
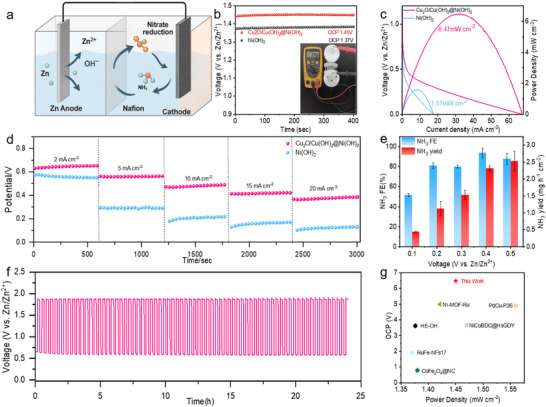
(a) Schematic diagram of a Zn‐NO_3_^−^ battery. (b) Open‐circuit voltage of Zn‐NO_3_^−^ cell assembled by Ni(OH)_2_ and Cu_2_O/Cu(OH)_2_@Ni(OH)_2_ electrodes. (c) Discharge curves and power density of Zn‐NO_3_^−^ batteries assembled with Ni(OH)_2_ and Cu_2_O/Cu(OH)_2_@Ni(OH)_2_ cathodes. (d) Discharge voltages at different current densities. (e) Ammonia yield rates and FEs of Zn‐NO_3_^−^ battery operated at different voltages. (f) Charge–discharge curves of Zn‐NO_3_^−^ battery at a constant current density of 2 mA cm^−2^. (g) Benchmarking of the Cu_2_O/Cu(OH)_2_@Ni(OH)_2_ catalyst's Zn‐NO_3_^−^ battery performance.

To demonstrate the practical utility of the Cu_2_O/Cu(OH)_2_@Ni(OH)_2_ heterostructure, we constructed a Zn‐NO_3_^−^ battery designed for simultaneous energy harvesting and environmental remediation. The schematic configuration of this device is depicted in Figure 6a. The battery constructed with the Cu_2_O/Cu(OH)_2_@Ni(OH)_2_ cathode and a 0.3 mm Zn anode delivers a high open‐circuit voltage (OCV) of 1.45 V, which is superior to the 1.37 V OCV obtained with the pristine Ni(OH)_2_ cathode (Figure 6b). This higher OCV confirms the enhanced catalytic activity endowed by the heterostructure. The power output and nitrate reduction performance of the batteries were then compared. As shown in Figure 6c, the battery equipped with the Cu_2_O/Cu(OH)_2_@Ni(OH)_2_ cathode delivered a peak power density of 6.47 mW cm^−2^. In stark contrast, the control device using a pristine Ni(OH)_2_ cathode only reached 1.57 mW cm^−2^. This represents a remarkable 4.1 fold enhancement in power density for our heterostructure‐based battery. This substantial improvement in power density is directly attributed to the unique synergistic interface of the heterostructure. As demonstrated in previous sections, the electron‐deficient Cu sites and large ECSA promote efficient NO_3_^−^ adsorption and activation, while the strong water dissociation capability ensures rapid proton supply. Together, these features significantly reduce the kinetic barriers for the NO_3_RR, leading to higher reaction rates and thus superior power output.

We further assessed the battery's ammonia synthesis capabilities under various discharge conditions. The device demonstrated remarkable stability, maintaining consistent discharge plateaus over a broad current density range from 2 to 20 mA cm^−2^ (Figure 6d). Particularly noteworthy is the performance at a low bias of 0.1 V. Under this condition, the cell achieved a high ammonia production rate of 2.78 mg h^−1^ cm^−2^ while sustaining an excellent FE of 93.6 % (Figure 6e). Furthermore, the battery demonstrates excellent long‐term stability, as evidenced by the stable galvanostatic cycling profile at 2 mA cm^−2^ for over 24 h (Figure 6f). To further highlight the advancement of our work, the key performance metrics of our Zn‐NO_3_^−^ battery were benchmarked against other state‐of‐the‐art systems reported in recent literature (Figure 6g; Table S2). Notably, the peak power density of 6.47 mW cm^−2^ achieved with our Cu_2_O/Cu(OH)_2_@Ni(OH)_2_ cathode is not only a significant leap over the control device but also ranks among the top‐tier values reported to date for aqueous Zn‐NO_3_^−^ batteries. This superior power output is a direct testament to the accelerated NO_3_RR kinetics and reduced overpotential enabled by the synergistic heterostructure interface, validating the effectiveness of our catalyst design strategy.

Collectively, these results underscore the catalyst's great promise for practical applications, enabling integrated nitrate removal, green NH_3_ synthesis, and efficient energy generation. Moreover, the exceptional performance of our Cu_2_O/Cu(OH)_2_@Ni(OH)_2_ cathode does more than just deliver high power density and ammonia yield. By operating at a significantly lower overpotential, it fundamentally alters the battery's operating conditions. This alleviates the harsh electrochemical environment imposed on the zinc anode, thereby creating a more favorable condition for its stable operation and potentially extending its cycle life [50, 51]. Furthermore, its high selectivity toward ammonia preempts the possibility of the destructive NO_2_^−^ shuttle effect, a critical step toward ensuring the long‐term chemical stability of the entire cell [26, 52]. While dedicated anode stabilization strategies remain essential for future development, our work demonstrates that designing a superior cathode is not just half the battle, but a critical prerequisite for enabling the long‐term viability of Zn‐NO_3_^−^ battery technology.

## 3 Conclusions

In summary, a wrinkled Cu_2_O/Cu(OH)_2_@Ni(OH)_2_ heterostructure was successfully engineered via a facile hydrothermal method. Comprehensive characterizations revealed that the incorporation of Cu fundamentally reshapes the pristine Ni(OH)_2_ by distorting its lattice, lowering crystallinity, and inducing a strong electronic coupling that drives electron transfer from Ni to Cu. This synergistic interplay forges a unique interfacial environment featuring electron‐deficient Cu^+^/Cu^2+^ centers adjacent to electron‐rich ─OH groups. When tested in an H‐type cell, the Cu_2_O/Cu(OH)_2_@Ni(OH)_2_ catalyst demonstrated exceptional performance, delivering a near‐unity FE of 99.6 % and a high NH_3_ yield of 1.14 mmol h^−1^ mgcat^−1^ at an applied potential of −0.6 V vs. RHE. This exceptional activity is further amplified in a flow electrolyzer, sustaining a 97.9 % FE with an industrially relevant yield of 17.13 mmol h^−1^ mgcat^−1^ at −600 mA cm^−2^. DFT calculations further confirmed that the hydrogenation reaction kinetics were more favorable than the competing HER. When employed as a cathode in a Zn‐NO_3_^−^ hybrid battery, the heterostructure‐based device delivered a high open‐circuit voltage of 1.45 V. Furthermore, it achieved a peak power density of 6.47 mW cm^−2^, which is four times greater than that of the control device. Beyond power generation, the integrated device also serves as a micro‐reactor for continuous ammonia synthesis. It produced NH_3_ at a steady rate of 2.78 mg h^−1^ cm^−2^ with an impressive 93.6 % FE. The device also demonstrated robust operational stability, exhibiting less than 2 % voltage decay over a 24 h period. These findings showcase a practical approach to synergistically couple energy generation with environmental remediation. This work not only presents a top‐tier electrocatalyst but also provides valuable insights into how interfacial engineering can be leveraged to design advanced materials for complex multi‐step reactions, highlighting a promising avenue for simultaneous green ammonia synthesis, nitrate remediation, and renewable electricity storage.

## Author Contributions

Taozhi Lv: Writing –original draft. Lekuan Yang: Investigation, Conceptualization. Can Hong: Methodology. Yihua Zhu: Software, Writing –review & editing. Jianhua Shen: Supervision, Writing –review & editing. Chunzhong Li: Resources, Funding acquisition.

## Conflicts of Interest

The authors declare no conflict of interest.

## Supporting information




**Supporting File**: advs73627‐sup‐0001‐SuppMat.docx.

## Data Availability

The data that support the findings of this study are available from the corresponding author upon reasonable request.
