# Supplementary material for: Electronic Interaction at Cu–O–Ni Heterointerface Promotes Electrocatalytic Nitrate Reduction to Ammonia and Zinc‐Nitrate Battery
Source: Adv Sci (Weinh). 2026 Jan 4;13(14):e21252. doi: 10.1002/advs.202521252 (PMC12970214; doi:10.1002/advs.202521252)
Supplement: Supplementary file 1 — Supporting File: advs73627‐sup‐0001‐SuppMat.docx. [file ADVS-13-e21252-s001.docx]

**Supporting Information**

**Electronic Interaction at Cu-O-Ni Heterointerface Promotes Electrocatalytic Nitrate Reduction to Ammonia and Zinc-Nitrate Battery**

Taozhi Lv, Lekuan Yang, Can Hong, Yihua Zhu, Jianhua Shen*, Chunzhong Li*

Shanghai Engineering Research Center of Hierarchical Nanomaterials, Key Laboratory for Ultrafine Materials of Ministry of Education, Frontiers Science Center for Materiobiology and Dynamic Chemistry, School of Materials Science and Engineering, East China University of Science and Technology, Shanghai 200237, China.

*Corresponding Author

Email: [jianhuashen@ecust.edu.cn](mailto:jianhuashen@ecust.edu.cn) (Jianhua Shen); [czli@ecust.edu.cn](mailto:czli@ecust.edu.cn) (Chunzhong Li).

1. **Expermental Section**

**Materials**

N-N dimethylformamide，Copper sulfate pentahydrate，potassium hydroxide，potassium nitrate were purchased from Aladdin Ltd (China). Nafion solution (5 wt%) were purchased from Alfa Aesar.All the reagents were used as received without further purification. Deionized (DI) water we used during experiment process.

**Synthesis of Ni(OH)_2_**

0.6 g of Ni(SO_4_)_2_ 6H_2_O was dispersed in 1.4 mL of deionized water, dissolved by ultrasonication, transferred to 50 mL of polytetrafluoroethylene liner (PTFE), and 34 mL of N,N dimethylformamide (DMF) was added, and the mounted high-pressure reactor was placed in an electrically heated constant-temperature blower drying oven, and the reaction temperature was set at 150 ℃, and the reaction was carried out for 24 h. After that, the supernatant was poured off, and the precipitate in the liner washed by deionized water and anhydrous ethanol for 2-3 times. The precipitate in the liner was washed by deionized water and anhydrous ethanol for 2-3 times, dried and ground to obtain Ni(OH)_2_ green powder.

**Synthesis of Cu_2_O/Cu(OH)_2_@Ni(OH)_2_**

0.6 g of Ni(SO_4_)_2_ 6H_2_O and 0.6 g of Cu(SO_4_) 5H_2_O were dispersed in 1.4 mL of deionized water, dissolved by ultrasonication, and then transferred to 50 mL of polytetrafluoroethylene liner (PTFE) with 34 mL of N,N dimethylformamide (DMF), and the mounted autoclave reactor was placed in the electrically heated thermostatic blast drying oven at a reaction temperature of 150 °C for 24 h. The supernatant was then poured off, and the precipitate in the liner was washed by deionized water and anhydrous ethanol 2-3 times. The reaction temperature was set at 150 ℃ for 24 h. Afterwards, the supernatant was poured off, and the precipitate in the liner was washed by deionized water and anhydrous ethanol for 2-3 times, and then grinded after drying to obtain the blue powder of Cu_2_O/Cu(OH)_2_@Ni(OH)_2_.

**Synthesis of CuNi alloy**

A certain amount of Cu_2_O/Cu(OH)_2_@Ni(OH)_2_ was taken and dispersed equally on the bottom of an alumina ark and placed in a tube furnace under an atmosphere of 5% H_2_/Ar, set the temperature increase rate of 5 °C min^-1^, and held the temperature at 250 °C for 4 h. After cooling down, a black powder of CuNi was obtained.

**Material Characterization**

***In situ* FTIR Spectrometry**

In situ FTIR measurements were performed by a Nicolet 5700 FTIR spectrophotometer in conjunction with a CHI 660 electrochemical workstation. The electrocatalyst was dropped onto the Au membrane and used as a working electrode for this experiment ,while Hg/HgO and graphite rod served as reference electrodes and counter electrode, respectively.In situ FTIR spectra were obtained for different electrolyte configurations when the electrodes were tested for LSV or constant potential.

**Scanning Electron Microscope (SEM)**

Scanning electron microscope (SEM) images were taken using a Zeiss Merlin microscope operated at 2 kV and equipped with an Inlens secondary electron detector. The powder samples after micro-milling were coated on conductive adhesive, placed on a test bench and put into the SEM instrument for observation of surface morphology.

**EPR Experiments**

5,5-dimethyl-1-pyrroline N-oxide (DMPO) was used to capture the instable hydrogen radical to form the DMPO-H adduct to generate EPR spectra. In the experiments, 5 ml electrolyte was mixed with 100 μL DMPO and was deoxygenated by bubbling Ar. The constant current electrolysis was carried out for 10 min in the H-type cell under the protection of Ar. EPR measurement was performed by 100G-18KG/EMX-8/2.7 spectrometer operating at a frequency near 9.5 GHz, sweep width of 200 G and power of 20 mW

**Electrochemical Measurements**

**Preparation of the Working Electrode.**

Typically, 20 mg of catalyst powder was dispersed in a mixed solvent containing 960 μL of ethanol and 40 μL of 5% nafion solution. After ultrasonic treatment for 20 min, the catalyst slurry was dropped uniformly onto a piece of CP with a size of 1 cm × 1 cm to obtain a working electrode. The loading was approximately 0.4 mg cm^-2^.

**Catalyst Activation Protocol:**

Catalyst Activation Prior to the electrochemical performance evaluation, a pre-activation process was conducted to stabilize the catalyst surface. The working electrode was subjected to continuous cyclic voltammetry (CV) scanning in the potential range of 0.6 V to -0.8 V vs. RHE at a scan rate of 100 mV s^-1^. The cycling was continued for approximately 200 cycles until the CV curves of consecutive cycles overlapped and reached a steady state, ensuring that the catalyst surface was dynamically stable and free from initial surface impurities.

**Electrochemical Nitrate Reduction Experiment.**

The electrochemical test was performed on a CHI 660e, electrochemical workstation, using an H-cell system separated by a Nafion 117 membrane. Catalyst-coated carbon paper, Ag/AgCl electrode were used as working, counter, and reference electrodes, respectively. The electrolyte used in this work was 1 M KOH solution (80 mL), which was evenly distributed to the cathodic and anodic chamber. KNO_3_ (100 mM) was added into the cathodic chamber as the reactant. Magnetic stirring was applied with a stirring rate of 500 rpm in the cathodic chamber during the electrochemical test. All potentials were recorded against the reversible hydrogen electrode (RHE) without special explanation. The linear sweep voltammetry (LSV) test was performed at a rate of 10 mV s^-1^, and hronopotentiometry test was conducted at each current for 1 h. Electrolytes from the cathode chamber were sampled before and after each NO_3_RR experiment for product analysis. The products were quantified using spectrophotometry and nuclear magnetic resonance hydrogen spectroscopy.

For each NO_3_RR reaction condition, triplicate experiments were performed, and the average and standard deviation were shown for all reported values. For all liquid samples from each NO_3_RR experiment, triplicate measurements were conducted, and the average was used in calculating performance metrics. Time-averaged nitrate removal rate, Faradaic efficiency (FE), and nitrogen selectivity (N-selectivity) were used as key metrics to assess NO_3_RR performance.

**Ion Concentration Detection Methods.**

The ion concentration was quantified by the UV-Vis spectrophotometer, after diluting to an appropriate concentration based on the calibration curves. The specific detection methods are as follows.

**Detection of nitrate-N.**

Firstly, a certain amount of electrolyte was extracted from the cathodic chamber and diluted to 5 mL to detection range. Then, 10 μL of 0.8 w% sulfamic acid solution and 0.1 mL of 1 M HCl were subsequently added into the above solution and mixed uniformity. After standing at room temperature for 20 min, the absorbance was recorded at a wavelength of 220 nm and 275 nm using an UV-Vis spectrophotometer. The final absorbance value was calculated according to the equation: A = A_220 nm_ - A_275 nm_. The concentration-absorbance curve was calibrated using the standard NaNO_3_ with different NO_3_^-^-N concentrations.

**Detection of nitrite-N.**

A mixture of p-aminobenzenesulfonamide (4g), N-(1-Naphthyl) ethylenediamine dihydrochloride (0.2 g), ultrapure water (50 mL) and phosphoric acid (10 mL, 1.70 g mL^-1^) was used as a color reagent. A certain amount of electrolyte was extracted from the cathodic chamber and diluted to 5 mL to detection range. Then, 0.1 mL of color reagent was added into the above solution and mixed uniformity. After standing at room temperature for 20 min, the absorbance was recorded at a wavelength of 540 nm. The concentration-absorbance curve was calibrated using the standard NaNO_2_ with different NO_2_^-^-N concentrations.

**Determination of ammonia-N.**

The amount of NH_3_ in the solution was determined by colorimetry using the indophenol blue method. The reagents required for the indophenol blue method are potassium sodium tartrate-salicylic acid solution, sodium hypochlorite solution, and sodium nitrosoferricyanide solution. Potassium sodium tartratesalicylic acid solution was configured by weighing 50 g of salicylic acid [C_6_H_4_(OH)COOH], adding about 100 mL of deionized water, then adding 160 mL of 2 M sodium hydroxide solution, followed by stirring for complete dissolution; then 50 g of potassium sodium tartrate (KNaC_4_H_6_O_6_·4H_2_O) was dissolved in water, and the above solution was combined into a 1000 mL volumetric flask and diluted with water to the standard line. Sodium hypochlorite solution was prepared by diluting with water and sodium hydroxide solution into sodium hypochlorite solution containing 3.5 g L^-1^ and 0.75 mol L^-1^ free base concentration. Sodium nitrosoferricyanide solution was prepared by weighing 0.1 g of sodium nitroso ferricyanide (Na_2_[Fe(CN)_5_NO]·_2_H_2_O) into a 10 mL colorimetric tube and adding water to the standard line. The usual method for sample determination was to take 20 μL of the reacted solution and add 200 μL of sodium nitrosoferricyanide solution, 2 mL of sodium potassium tartrate solution-salicylic acid and 1 mL of sodium hypochlorite solution, followed by dilution with water to 10 mL. After color development for 60 min, the absorbance was then measured at 655 nm using a 10 mm cuvette with water as reference. A standard curve was established with a series of standard concentrations of ammonium chloride solution.

**Isotope Labeling Experiments**

99 % Na^15^NO_3_ was used as the feeding N-source to perform the isotopic labeling nitrate reduction experiments, to clarify the source of ammonia. 1 M KOH solution was used as the electrolyte and Na^15^NO_3_ with a concentration of 0.1M ^15^NO_3_^-^ (^15^N) was added into the cathode chamber as the reactant. After electroreduction, electrolyte with obtained ^15^NH_4_ ^+^ (^15^N) was taken out and the pH value was adjusted to be weak acid with 1M H2SO4 for further quantification by 1H NMR (600 MHz) with external standards of maleic acid. The calibration curve was created as follows. First, a series of ^15^NH^4 +^ ^15^N solutions ((^15^NH_4_)_2_SO_4_)) with known concentrations (1, 2, 5, 10, 20 mM) were prepared in 1 M KOH solution as standards. Second, 50 mL of the ^15^NH_4_ ^+^ (^15^N) standard solution with different concentrations was mixed with 0.02 g maleic acid. Third, 100 μL deuterium oxide (D_2_O) was added in 0.5 mL above mixed solution for the NMR detection. Fourth, the calibration was realized using the peak area ratio between ^15^NH_4_ ^+^ (^15^N) and maleic acid because the ^15^NH4 ^+^( ^15^N) concentration and the area ratio were positively correlated. Similarly, the amount of ^14^NH4 ^+^ ( ^14^N) was quantified by this method when Na^14^NO_3_ was used as the feeding N source

**Calculations of Nitrate Conversion, Selectivity, NH_3_ Yield Rate, and Faradaic Efficiency**

The conversion rate was calculated according to the Eq. S1:

Conversion = Δc_NO3_-/c_0_ × 100% (Eq. S1)

The selectivity to ammonia and nitrite were obtained by the Eq. S2-3:

S_NH3_ = c_NH3_/Δc_NO3_- × 100% (Eq. S2)

S_NO2_ - = c_NO2_-/Δc_NO3_ - × 100% (Eq. S3)

The FE was defined from the charge consumed for NH_3_ synthesis and total charge

passed through the electrode:

FE = (8F × c_NH3_ × V) / (M_NH3_ × Q) (Eq. S4)

The yield rate was obtained by the Eq. S5:

Yield_NH3_ = (c_NH3_ × V) / (M_NH3_ × t × S) (Eq. S5)

Where Δc_NO3_^-^ is the concentration difference of NO_3_^-^ before and after electrolysis, c_0_ is the initial concentration of NO_3_^-^ , c_NO3_- is the mass concentration of NO_3_^-^, c_NH3_ is the produced mass concentration of NH_3_, c_NO2_^-^ is the produced mass concentration of NO_2_^-^, M_NH3_ is the molar mass of NH_3_, F is the Faradaic constant (96485 C mol^-1^ ), Q is the quantity of applied electricity, V is the volume of electrolyte in the cathode chamber (40 mL), it is the electrolysis time (1 h), S is the geometric area of the working electrode (1 cm^2^ ).

**Calculation details**

All the DFT calculations were conducted using the Dmol3 module of Materials Studios 2017. The electronic exchange and related energy were treated using the Perdew, Burke and Ernzerhof (PBE) functional within the generalized gradient approximation (GGA). The conductor like screening model (COSMO) method was adopted to consider the electrostatic interaction of adsorbate and water solvent. The DFT semi-core pseudo potentials (DSPPs) core treatment with the relativistic effects were implemented to consider the core-electron (e^-^) interaction, which superseded core e- by a single valid electric-potential to simplify the calculations. The numerical basis set of double numerical plus polarization (DNP) was utilized. Thermal smearing of orbital occupation is set to 0.005 Ha (1 Ha = 27.21 eV). The SCF density convergence tolerance was 1×10^-5^ Ha. The maximum force, displacement, and energy of geometry optimal convergence tolerance are 0.004 Ha/Å, 0.005 Å, and 2×10^-5^ Ha. The standard hydrogen electrode (SHE) model was calculated the Gibbs free energy change (ΔG) for each basic step. Based on this method, the ΔG value can be determined as follows:

ΔG = ΔE + ΔZPE – TΔS (Eq. S6)

where ΔE is the adsorption energy, ΔZPE is the zero-point energy difference and TΔS is the entropy difference between the gas phase and adsorbed state. The adsorption free energy of NO_3_^‒^ (ΔG(*NO_3_)) was calculated with assistance of the gaseous HNO_3_ as follows:

ΔG(*NO_3_) = G(*NO_3_) ‒ G(*) ‒ [G(HNO_3_) ‒ 0.5 × G(H_2_)] + ΔG_correct_ (Eq. S7)

where G(*) and G(*NO_3_) are the Gibbs free energies of the bare catalyst and that with the adsorbed NO_3_^‒^, respectively. G(HNO_3_) and G(H_2_) represent the Gibbs free energy.

1. **Supporting Figures**


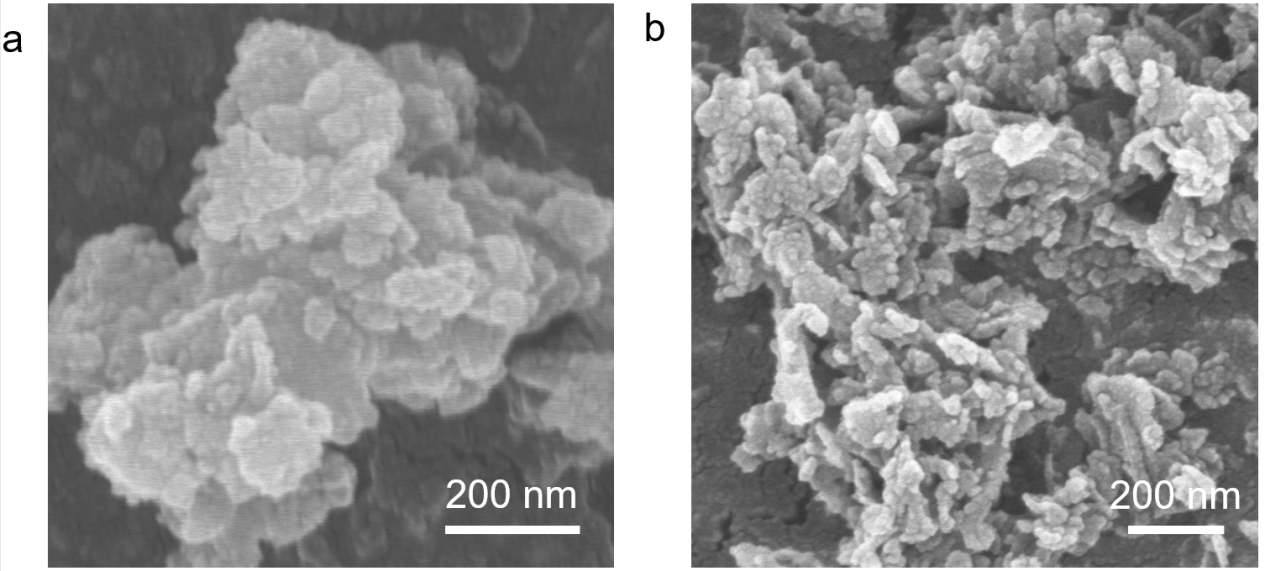


**Figure S1.** SEM images of (a) Cu_2_O/Cu(OH)_2_@Ni(OH)_2_ and (b) Ni(OH)_2_.

**Figure S2.** Full XPS spectrum of Cu_2_O/Cu(OH)_2_@Ni(OH)_2_.

**
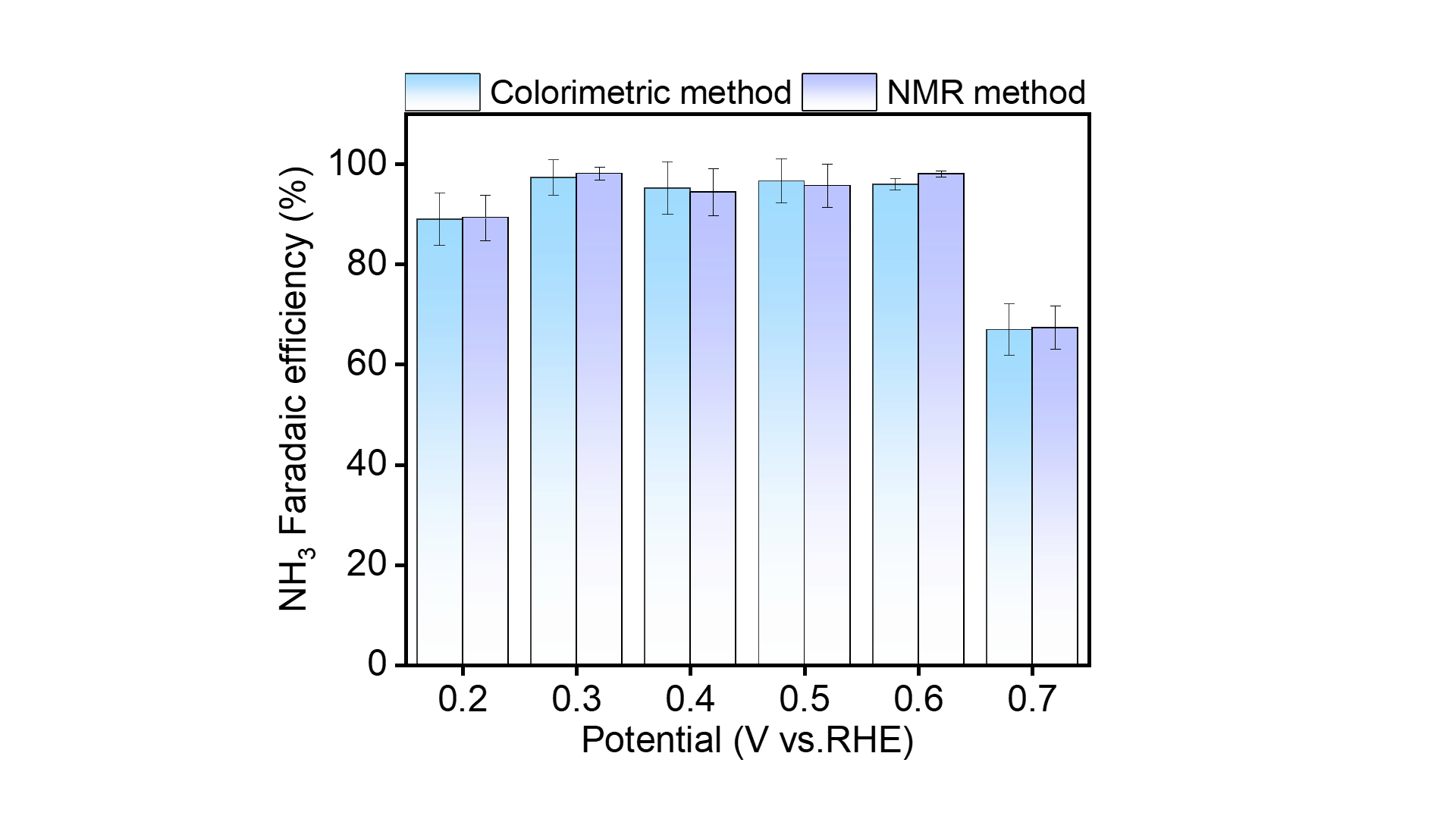
**

**Figure S3**. Comparison of the FE of ammonia synthesis determined by the indophenol blue colorimetric method and the ^1^H NMR method at various potentials.


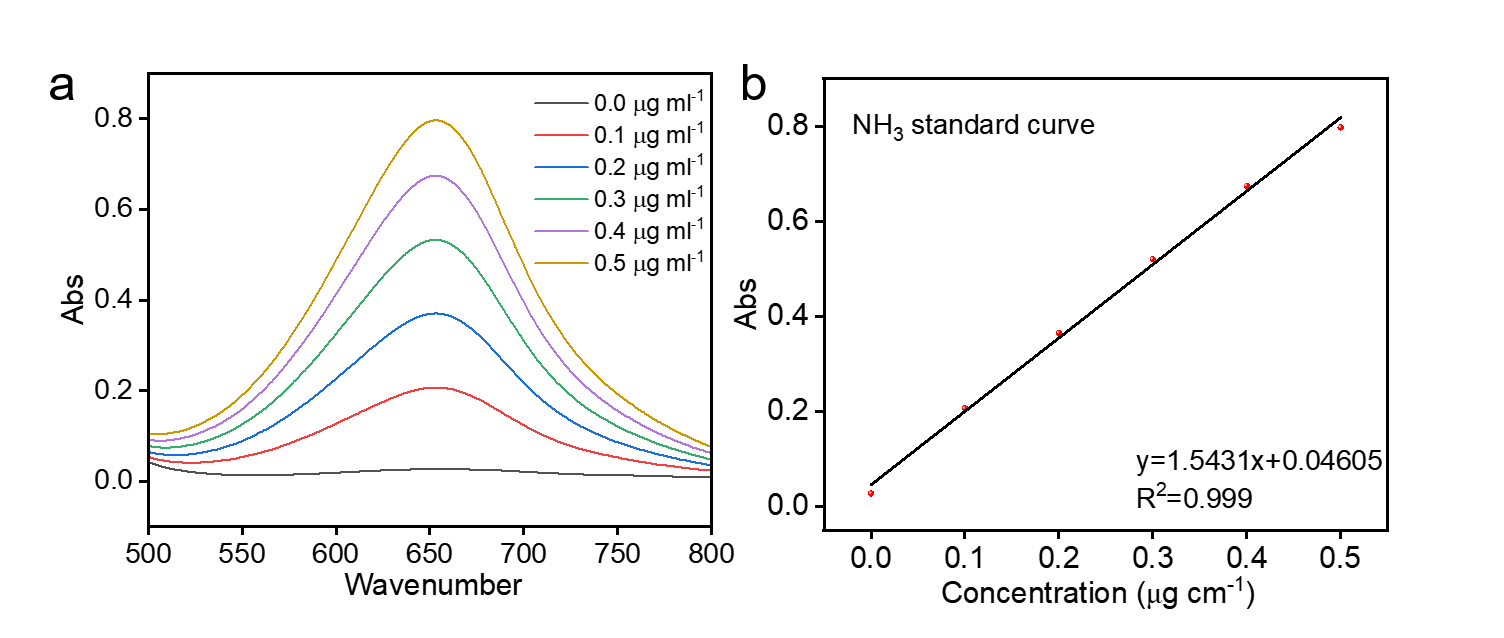


**Figure S4.** (a) UV-vis absorption spectra of NH_4_^+^-N assays after incubated for 2 h at ambient conditions. (b) Calibration curve used for the calculation of NH_3_ concentrations.


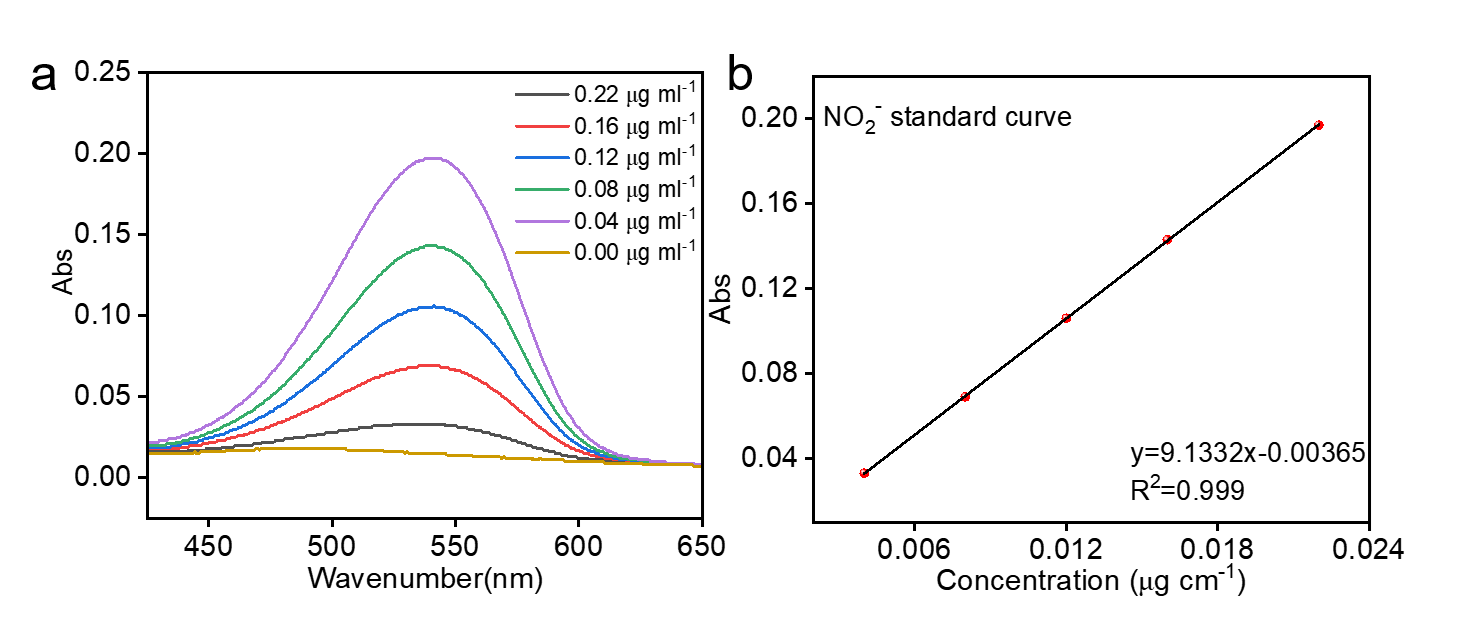


**Figure S5.** (a) UV-vis absorption spectra of NO_2_^-^ assays after incubated for 20 min at ambient conditions. (b) Calibration curve used for calculation of NO_2_^-^ concentrations.


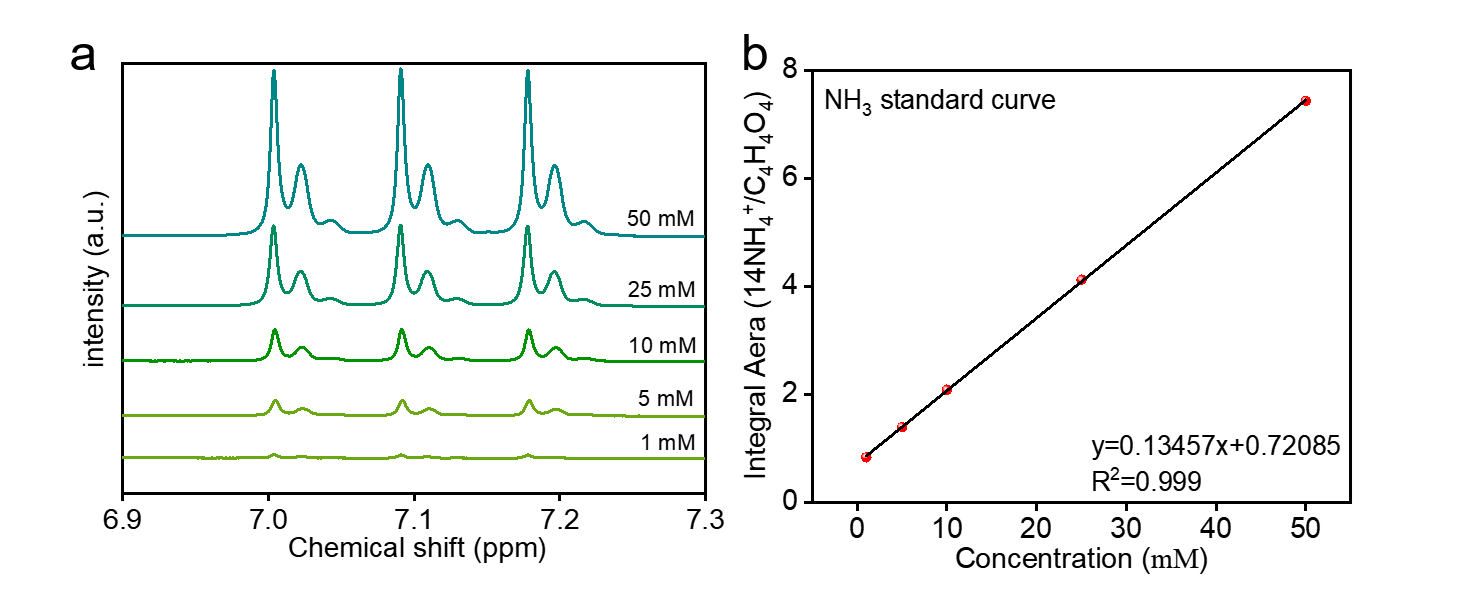


**Figure S6.** (a) ^1^H NMR spectra of ^14^NH_4_^+^ standard samples with different concentrations and (b) corresponding calibration curves.


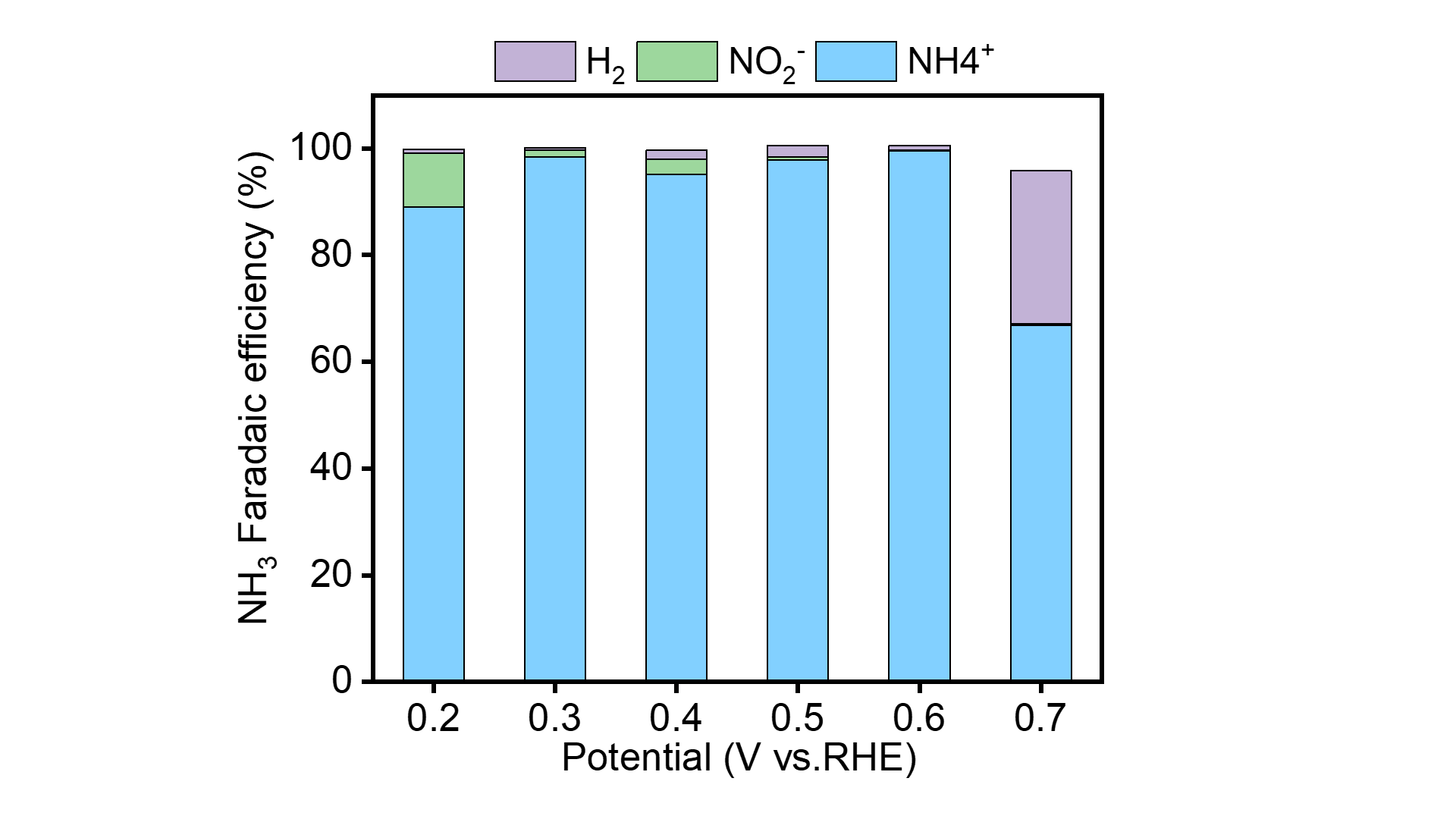


**Figure S7.** FE distribution of NH_3_, NO_2_^-^, and H_2_ products for the Cu_2_O/Cu(OH)_2_@Ni(OH)_2_ catalyst at different applied potentials.


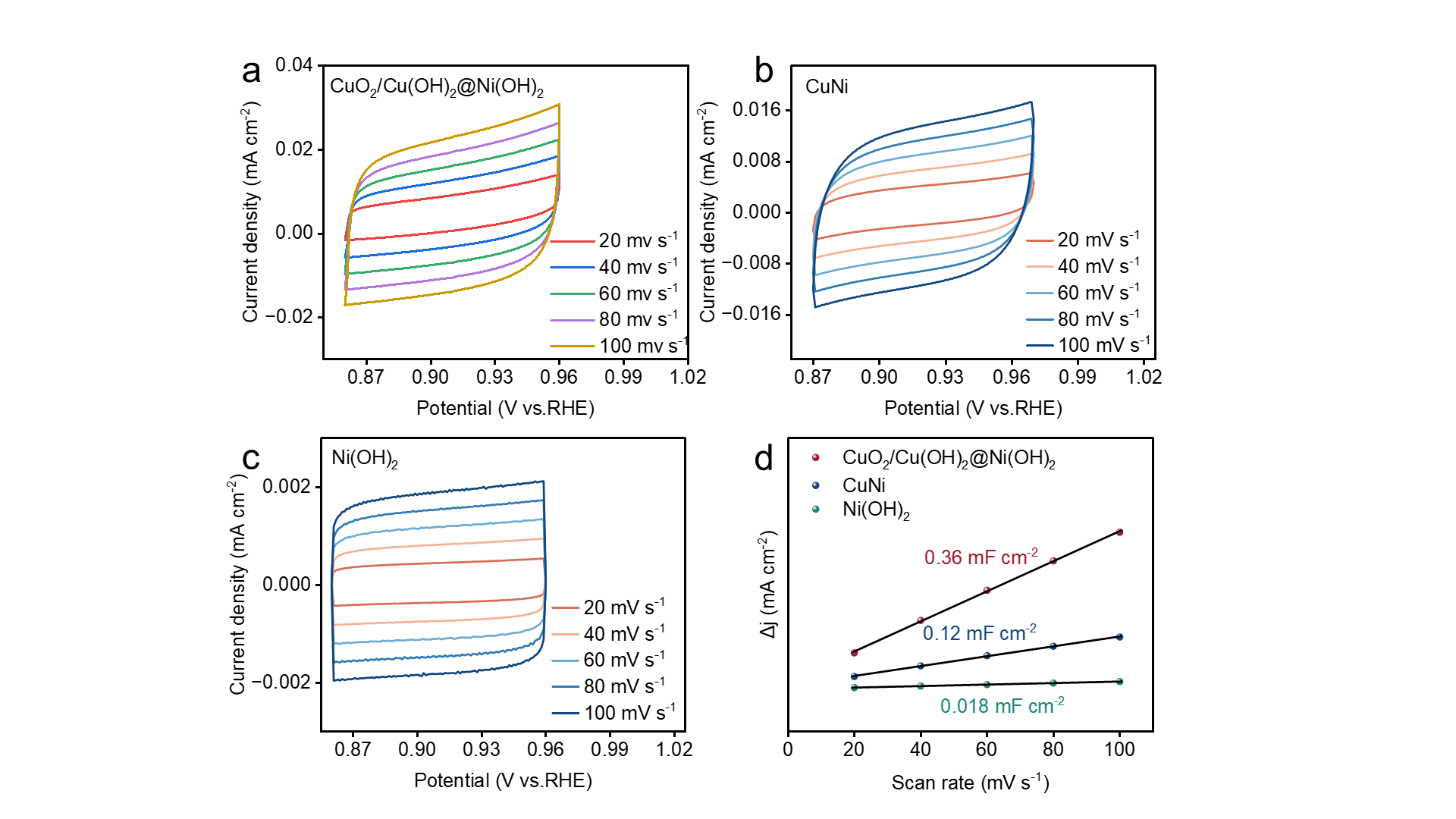


**Figure S8.** CV curves of (a) Cu_2_O/Cu(OH)_2_@Ni(OH)_2_, (b) CuNi and (c) Ni(OH)_2_ at different scanning rates. (d) Linear fit plot of current density difference relative to sweep speed, slope representing C_dl_.


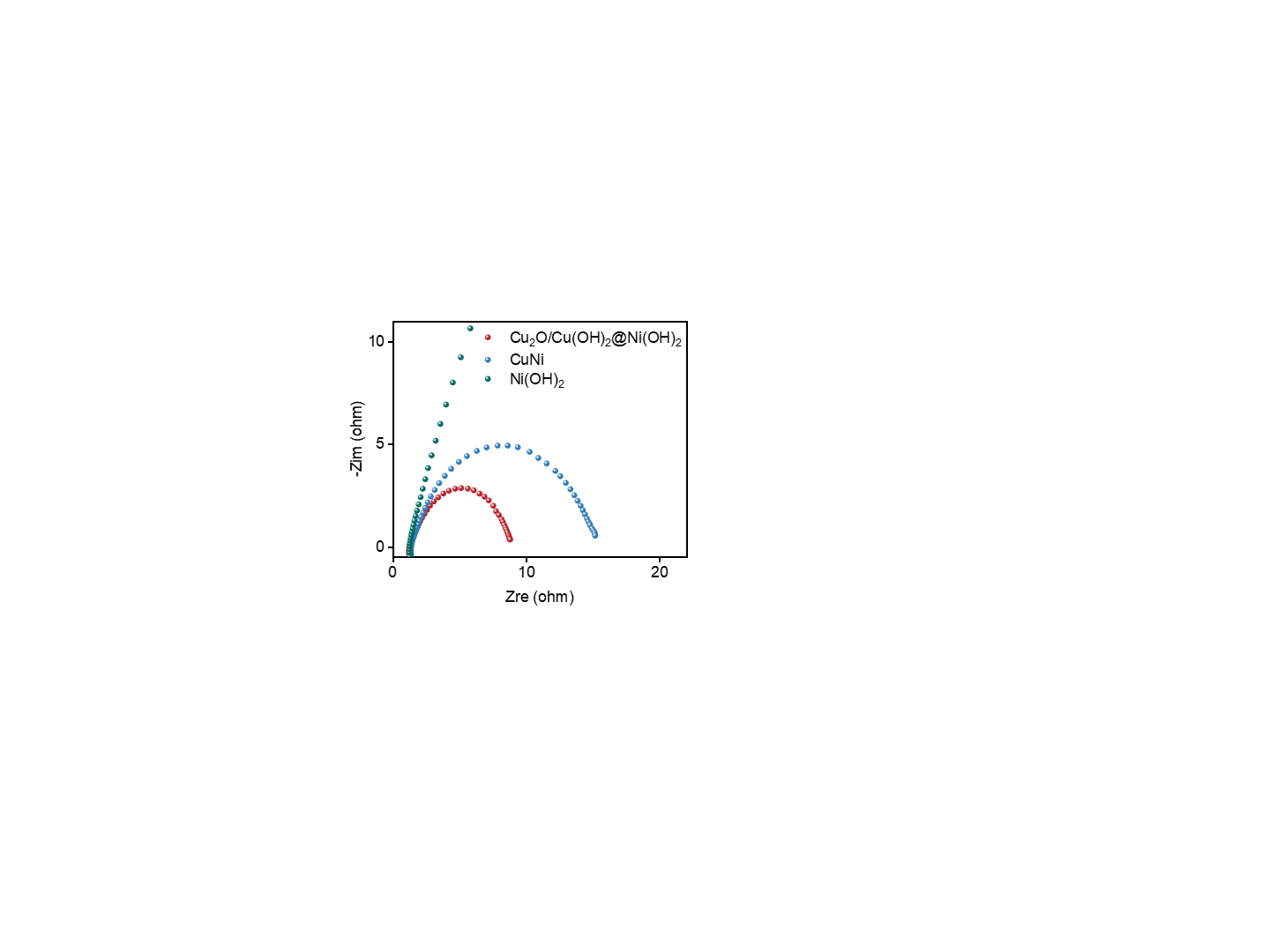


**Figure S9.** Nyquist impedance diagram of Cu_2_O/Cu(OH)_2_@Ni(OH)_2_, CuNi and Ni(OH)_2_ catalysts.


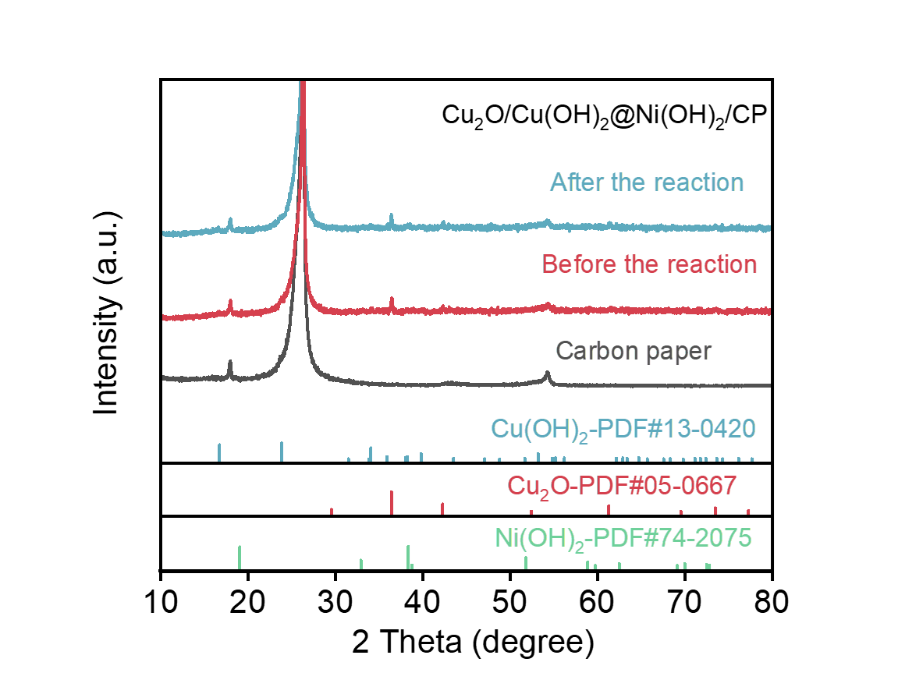


**Figure S10.** XRD of Cu_2_O/Cu(OH)_2_@Ni(OH)_2_ catalyst on carbon paper after stability test.


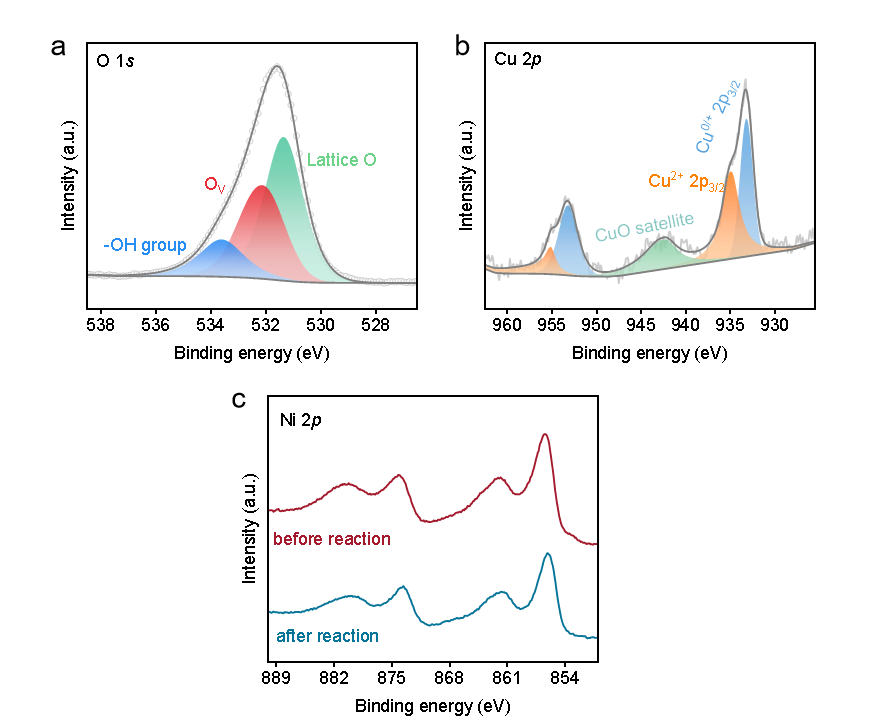


**Figure S11.** XPS of O 1*s* (a), Cu 2*p* (b) and Ni 2*p* (c) in Cu_2_O/Cu(OH)_2_@Ni(OH)_2_ catalyst after stability test.


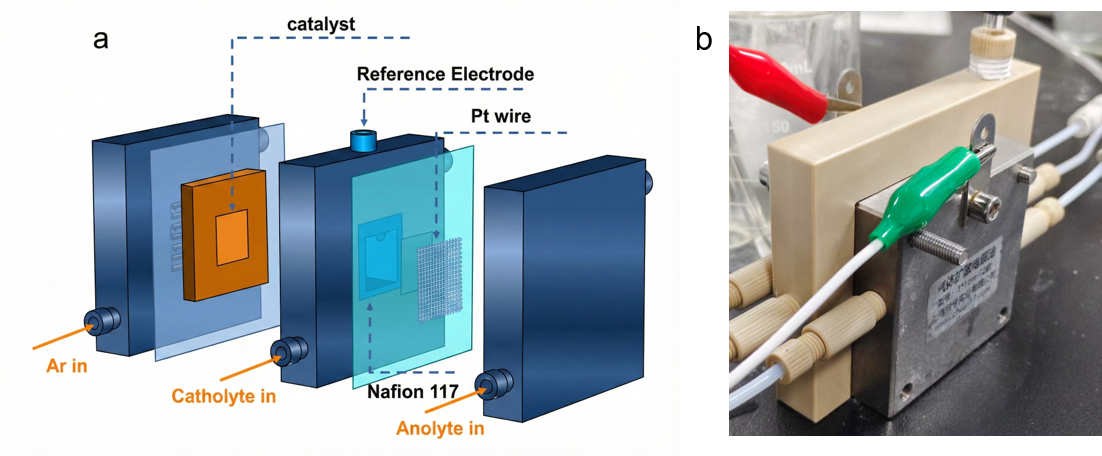


**Figure S12.** Configuration of the flow cell setup. (a) Schematic illustration of the internal structure of the flow cell, showing the cathode/anode chambers and membrane assembly. (b) Digital photograph of the assembled flow cell reactor.


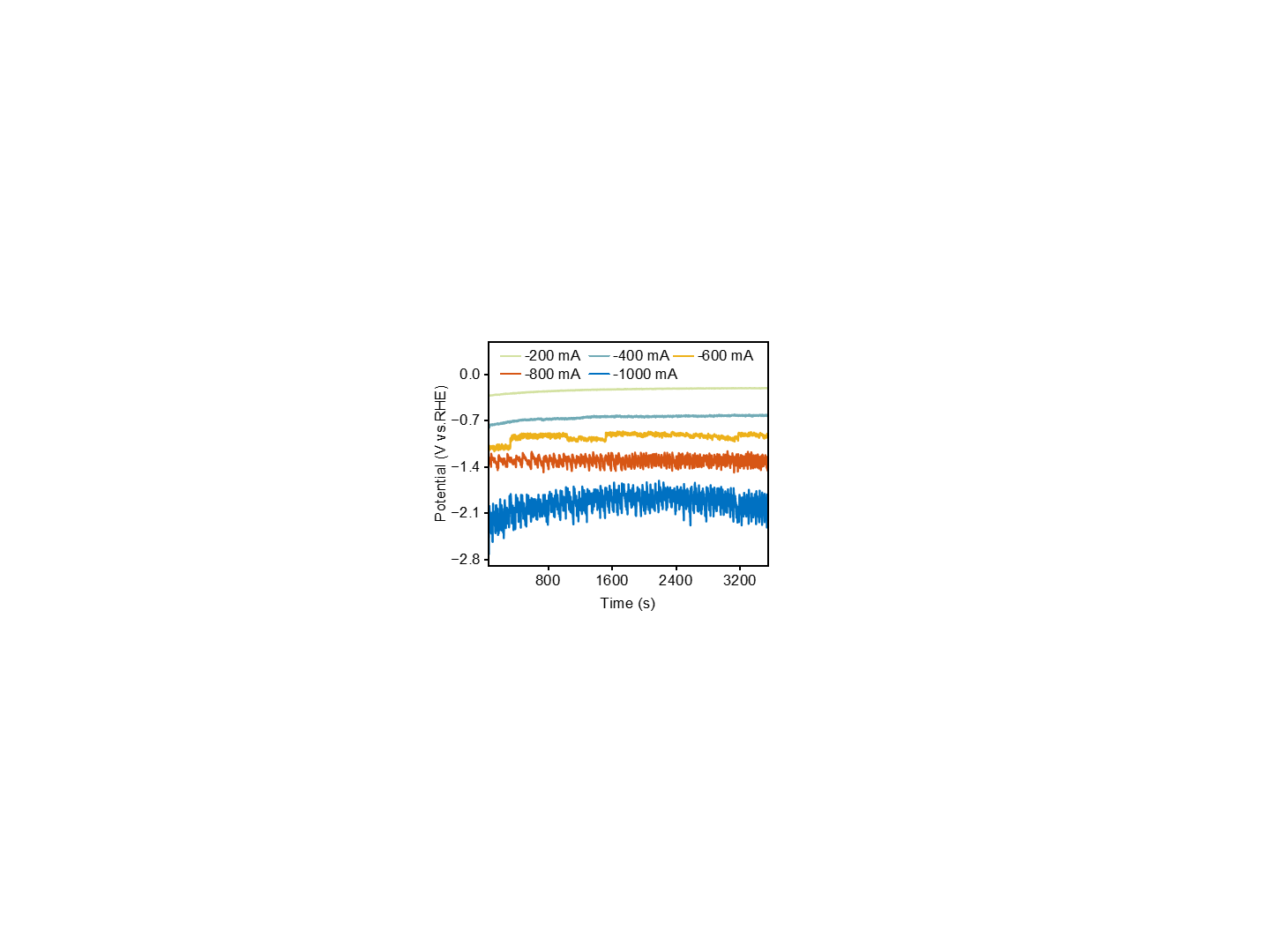


**Figure S13.** Real-time spectrum of potential of Cu_2_O/Cu(OH)_2_@Ni(OH)_2_ catalyst at different current densities.


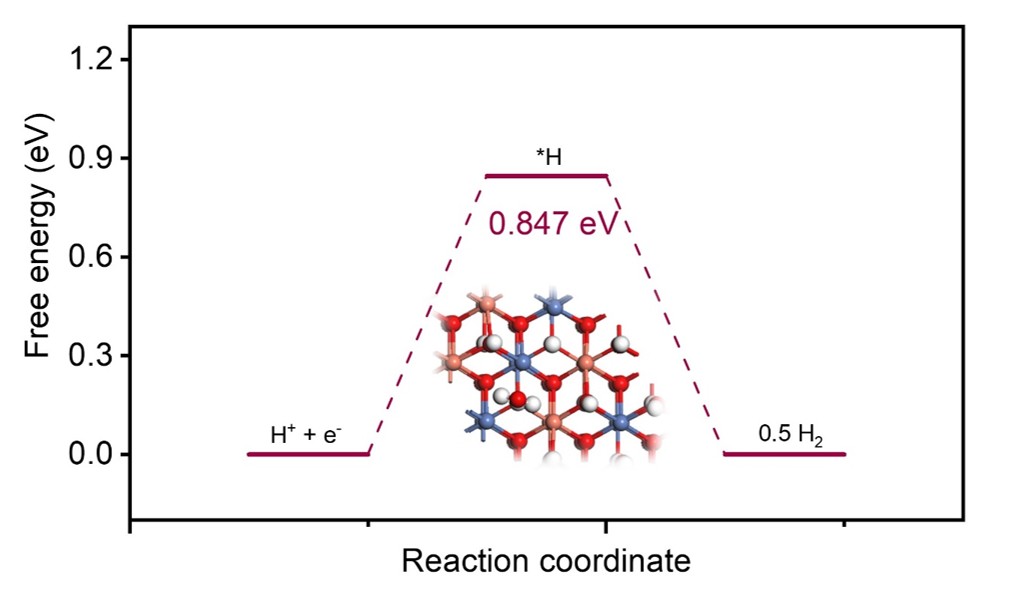


**Figure S14.** Free energy diagram of hydrogen adsorption on Cu_2_O/Cu(OH)_2_@Ni(OH)_2_ surface.

1. **Supporting Tables**

**Table S1.** Comparison with recently reported catalysts of electrocatalytic nitrate reduction performance.

| **Catalysts** | **Electrolyte** | | **NH_3_ Yield rate (mg h^-1^ mg_cat_^-1^)** | **FE (%)** | **Ref** |
| --- | --- | --- | --- | --- | --- |
| Cu_2_O/Cu(OH)_2_@Ni(OH)_2_ | 1 M KOH  +0.1 M KNO_3_ | **19.4** | | **99.6** | **This work** |
| Cu/Cu_2_O | 0.5 M Na_2_SO_4_  +200 ppm NO_3_-N | 4.16 | | 95.8 | [1] |
| CuO-Co_3_O_4_/Ti | 0.1 M Na_2_SO_4_  +50 ppm NO_3_-N | 1.65 | | 54.5 | [2] |
| CoNiO₂ @ NF | - 1. M NaOH   + 500 ppm NO_3_-N | 4.19 | | 97.7 | [3] |
| Ni-CuO | 0.5 M Na_2_SO_4_  +0.10 M KNO_3_ | 16.0 | | 95.26 | [4] |
| LaNi_0.2_Cu_0.8_O_3-δ_ | 0.1 M KOH  +0.1 M KNO_3_ | 5.33 | | 88.9 | [5] |
| FeCo/ Sm_0.9_FeO_3_ | - 1. M PBS   + 0.1 M KNO_3_ | 17.2¹ | | 90.3 | [6] |
| Cu-B_x_ | 0.5 M KOH  + 0.05 M KNO₃ | 16.9 | | 94 | [7] |
| Cu-SAC | 0.05 M Na_2_SO_4_  +500 ppm NO_3_-N | 0.585 | | 46.7 | [8] |
| CuCl-BEF | 0.5 M Na_2_SO_4_  +100 ppm NO_3_-N | 2.15 | | 44.7 | [9] |
| Co/CoO NSA | - 1. M Na_2_SO_4_   +200 ppm NO_3_-N | 3.31 | | 93.8 | [10] |
| Pd/TiO_2_ | 1 M LiCl  + 0.25 M LiNO_3_ | 1.12 | | 92.1 | [11] |

**Table S2.** Comparison with recently reported catalysts of Zn-NO_3_^-^ battery performance.

| **Catalysts** | **Electrolyte** | | **OCV**  **(V vs.**  **Zn/Zn^2+^)** | **Power density**  **(mW cm^−2^)** | **Ref** |
| --- | --- | --- | --- | --- | --- |
| Cu_2_O/Cu(OH)_2_@Ni(OH)_2_ | 1 M KOH  +0.1 M KNO_3_ | **1.45** | | **6.47** | **This work** |
| CoFe_2_O_4_@NC | 1 M KOH  +0.1 M  NO3 | 1.38 | | 0.78 | [12] |
| RuFe-NFs17 | 0.5 M  Na_2_SO_4_+ 0.1 M NO_3_^-^ | 1.37 | | 1.9 | [13] |
| PdCu-P26 | - 1. M KOH   +0.01 M NO_3_^-^ | 1.56 | | 4.9 | [14] |
| NiCoBDC@HsGDY | 1 M KOH+0.1 M NO_3_^–^ | 1.47 | | 3.66 | [15] |
| HE-OH | 1.0 M KOH+1.0 M KNO_3_ | 1.376 | | 3.62 | [16] |
| Ni-MOF-Ru | 6.0 M KOH+1.0 M KNO_3_ | 1.421 | | 4.99 | [17] |

**Reference:**

1. Wang, Y., et al., *Unveiling the Activity Origin of a Copper-based Electrocatalyst for Selective Nitrate Reduction to Ammonia.* Angewandte Chemie International Edition, 2020. **59**(13): p. 5350-5354.
2. Gao, J., et al., *Non-precious Co_3_O4-TiO_2_/Ti cathode based electrocatalytic nitrate reduction: Preparation, performance and mechanism.* Applied Catalysis B: Environmental, 2019. **254**: p. 391-402.
3. Zhang, Y., et al., *Electrocatalytic nitrate reduction to ammonia by sea-urchin-like CoNiO_2_ under mild conditions.* Cell Reports Physical Science, 2024. **5**(6).
4. Li, Y., et al., *Hollow Square Ni-Doped Copper Oxide Catalyst Boosting Electrocatalytic Nitrate Reduction.* ACS Catalysis, 2025. **15**(3): p. 1672-1683.
5. Zhang, W., et al., *LaNi0.2Cu0.8O3-δ Perovskites as Electrocatalysts for Electro-synthesis of Ammonia from Nitrogen Oxyanion.* ChemCatChem, 2024. **16**(15): p. e202400185.
6. Hu, P., et al., *In-situ exsolution of FeCo nanoparticles over perovskite oxides for efficient electrocatalytic nitrate reduction to ammonia via localized electrons.* Applied Catalysis B: Environment and Energy, 2024. **357**: p. 124267.
7. Ma, H., et al., *Dendritic copper oxide catalyst engineering weak-polarity Cu-O bond for high-efficiency nitrate electroreduction.* Journal of Hazardous Materials, 2024. **470**: p. 134261.
8. Zhu, T., et al., *Single-Atom Cu Catalysts for Enhanced Electrocatalytic Nitrate Reduction with Significant Alleviation of Nitrite Production.* Small, 2020. **16**(49): p. e2004526.
9. Sun, W.-J., et al., *Built-in Electric Field Triggered Interfacial Accumulation Effect for Efficient Nitrate Removal at Ultra-Low Concentration and Electroreduction to Ammonia.* Angewandte Chemie International Edition, 2021. **60**(42): p. 22933-22939.
10. Yu, Y., et al., *Promoting selective electroreduction of nitrates to ammonia over electron-deficient Co modulated by rectifying Schottky contacts.* Science China Chemistry, 2020. **63**(10): p. 1469-1476.
11. Guo, Y., et al., *Pd doping-weakened intermediate adsorption to promote electrocatalytic nitrate reduction on TiO2 nanoarrays for ammonia production and energy supply with zinc–nitrate batteries.* Energy & Environmental Science, 2021. **14**(7): p. 3938-3944.
12. Lee, Y., Theerthagiri, J., Yodsin, N., Min, A., Moon, C.J., Jungsuttiwong, S., and Choi, M.Y. (2024). *Mitigating intraphase catalytic-domain transfer via CO_2_ laser for enhanced nitrate-to-ammonia electroconversion and Zn-nitrate battery behavior*. Angew Chem. Int. Ed. 63, e202416807. 10.1002/anie.202416807.
13. Wang, Y., Sun, M., Zhou, J., Xiong, Y., Zhang, Q., Ye, C., Wang, X., Lu, P., Feng, T., Hao, F., et al. (2023). *Atomic coordination environment engineering of bimetallic alloy nanostructures for efficient ammonia electrosynthesis from nitrate*. Proc. Natl. Acad. Sci. U.S.A. 120, e2306461120. 10.1073/pnas.2306461120.
14. Min, X., and Liu, B. (2023). *Microenvironment engineering to promote selective ammonia electrosynthesis from nitrate over a PdCu hollow catalyst*. Small 19, 2300794. 10.1002/smll.202300794.
15. Jiahao Ma, Yuting Zhang, Biwen Wang, Zixin Jiang, Qiuyu Zhang, and Sifei Zhuo. *Interfacial Engineering of Bimetallic Ni/Co-MOFs with H-Substituted Graphdiyne for Ammonia Electrosynthesis from Nitrate*. ACS Nano 2023 17 (7), 6687-6697. DOI: 10.1021/acsnano.2c12491
16. Yao, Y., et al., *Regulating the d-Band Center of Metal–Organic Frameworks for Efficient Nitrate Reduction Reaction and Zinc-Nitrate Battery*. ACS Catalysis, 2024. 14(21): p. 16205-16213.
17. Chen, M., et al., *A zinc-nitrate battery for efficient ammonia electrosynthesis and energy output by a high entropy hydroxide catalyst*. Chinese Chemical Letters, 2025: p. 111294.
